# Analysis of Terpene Synthase Family Genes in *Camellia sinensis* with an Emphasis on Abiotic Stress Conditions

**DOI:** 10.1038/s41598-020-57805-1

**Published:** 2020-01-22

**Authors:** Han-Chen Zhou, Lubobi Ferdinand Shamala, Xing-Kai Yi, Zhen Yan, Shu Wei

**Affiliations:** 1 State Key Laboratory of Tea Plant Biology and Utilization, Anhui Agricultural University, Hefei, China; 2 Tea Research Institute, Anhui Academy of Agricultural Sciences, Huangshan, China; 3 Horticultural Institute, Anhui Academy of Agricultural Sciences, Hefei, China

**Keywords:** Plant stress responses, Secondary metabolism

## Abstract

For a better understanding terpenoid volatile production in *Camellia sinensis*, global terpenoid synthase gene (*TPS*) transcription analysis was conducted based on transcriptomic data combined with terpenoid metabolic profiling under different abiotic stress conditions. Totally 80 *TPS*-like genes were identified. Twenty-three *CsTPS* genes possessed a complete coding sequence and most likely were functional. The remaining 57 in the currently available database lack essential gene structure or full-length transcripts. Distinct tempo-spatial expression patterns of *CsTPS* genes were found in tea plants. 17 genes were substantially expressed in all the tested organs with a few exceptions. The other 17 were predominantly expressed in leaves whereas additional eight were primarily expressed in flowers. Under the treatments of cold acclimation, salt and polyethylene glycol, *CsTPS67*, *-69* and -*71* were all suppressed and the inhibited expression of many others were found in multiple stress treatments. However, methyl jasmonate resulted in the enhanced expression of the majority of *CsTPS* genes. These transcription data were largely validated using qPCR. Moreover, volatile terpenoid profiling with leaves, flowers and stress-treated plants revealed a general association between the abundances of mono- and sesqui-terpenoids and some *CsTPS* genes. These results provide vital information for future studies on *CsTPS* regulation of terpenoid biosynthesis.

## Introduction

Plant terpenoids (isoprene-C5, monoterpenes-C10, sesquiterpenes-C15, diterpenes-C20, and polyterpenoids-C5xn) possess diverse functions in plant growth and development^1–7^. They play significant ecological roles in the interactions between plants and stress conditions. Generally, terpenoid molecules smaller than diterpenoids are volatile and well known for their airborne signaling function, particularly against herbivore attack^8,9^. High volatility of monoterpenes and sesquiterpenes enhances the flavor and aroma of crop products^10^ such as tea, which is a popular beverage well known for its fragrance and aroma^11^. Tea volatile terpenoids not only are defense components against insects^12^ or high solar radiation^13^, but are also essential odorants of tea products with a direct influence on flavor and quality^14–16^. Aroma from volatile terpenoids is one of the main sensory properties affecting tea flavor quality^17^. For instance, monoterpene alcohols such as linalool and geraniol, two of the most abundant and odor active terpenoids in tea^15^, impart pleasant floral scent to green tea and black tea^17^.

**Figure 1:**
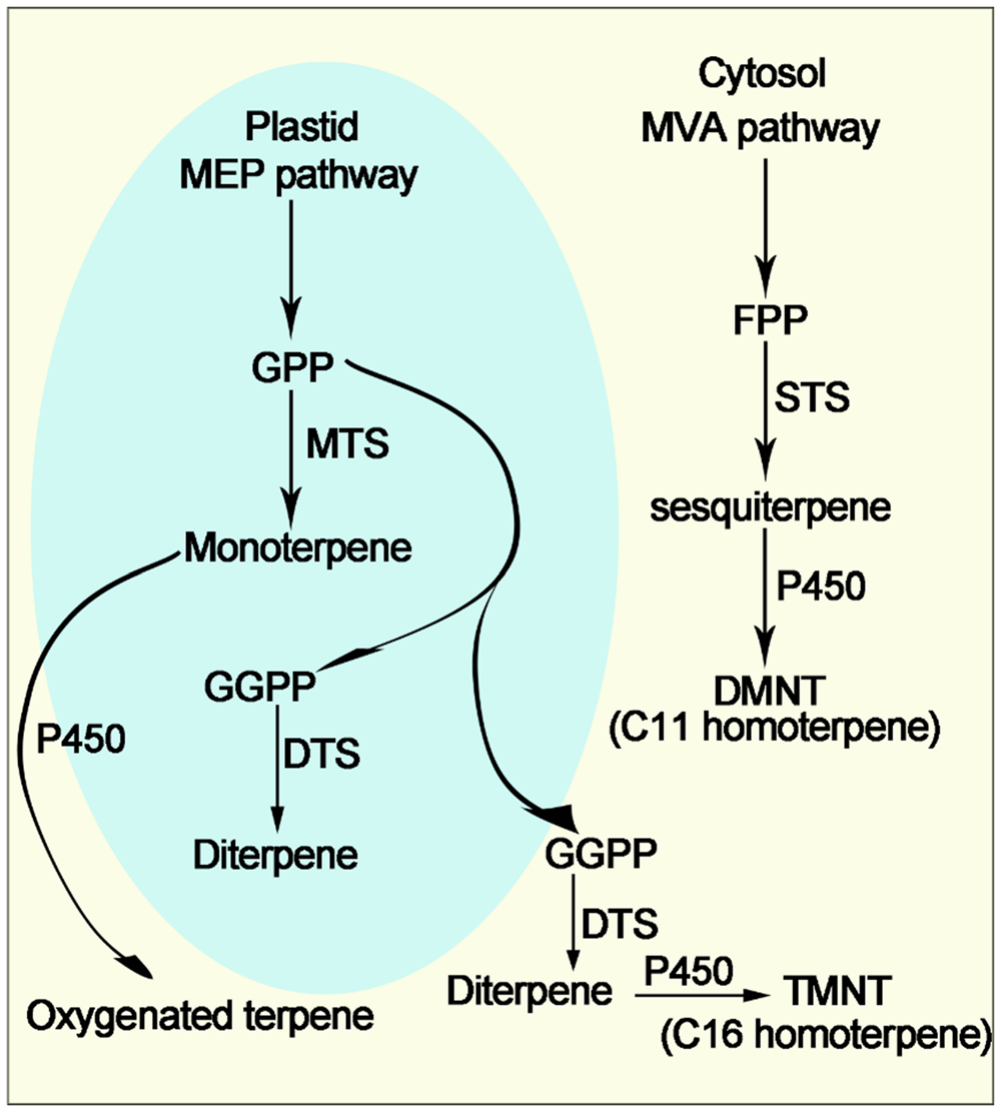
The pathway of terpene synthase gene responsible for the formation of terpenoids in planta. MTS, monoterpene synthase; STS, sesquiterpene synthase; DTS, diterpene synthase.

Terpene synthases possess a characteristic catalytic function that generates multiple terpenoid products with one substrate^18^, thus collectively contributing to numerous and different structures of plant terpenoids in addition to other modifying enzymes such as uridine diphosphate (UDP)-glucosyl transferases^19,20^ and P450s^21^. TPSs are responsible for converting the precursors of geranyl diphosphate (GPP), isoprenyl diphosphate (IPP), farnesyl diphosphate (FPP) and geranylgeranyl diphosphate (GGPP) into a multitude of cyclic and acyclic monoterpenes, sesquiterpenes and diterpenes by different pathways, respectively (Fig. 1)^18,22^. In general, the TPS family is characterized by two large domains defined in the PFAM (Protein families) database (pfam.xfam.org/): PF01397 corresponds to the N-terminal region and PF03936 corresponds to the C-terminal metal cofactor binding domain^23^. In addition, the expected gene size and organization comply with seven exons for TPS-a, TPS-b, and TPS-g and between 13 and 15 exons for TPS-e/TPS-f and TPS-c^24,25^. TPSs also contain structural features such as the conserved ‘DDXXD’ and ‘NSE’ motifs^24^.

The majority of sequenced plant genomes that have been analyzed contain different sizes of *TPS* families with 30 to 100 members, which probably evolved through duplication of genes followed by functional divergence^24^. The plant TPSs are divided into six subfamilies named from TPS-a to TPS-f based on their amino acid sequence relatedness^26^ therein TPS-a, TPS-b, and TPS-g are angiosperm-specific clades while TPS-d are gymnosperm-specific clade. TPS-h is specific to the spike moss and TPS-e and -f are proposed to combined together into the group of TPS-e/f^24^. So far the *TPS* gene family members have been characterized in many plant species including *Arabidopsis thaliana*^22,24^, *Sorghum bicolor* L.^27^, grape (*Vitis vinifera*)^23^, tomato (*Solanum lycopersicum*)^28^, apple (*Malus domestica*)^25^, poplar (*Populus trichocarpa*)^29^, *Eucalyptus* species^30^ and carrot (*Daucus carota* L.)^31^.

Compared to other plants, the tea plant is a perennial woody plant species belonging to the *Theaceae* family with a characteristic secondary metabolite profile containing approximately 100 types of different volatile terpenoids^32^. To date, only few tea *TPS* genes have been identified. A striking study presents *CsLIS/NES*, which generates two splicing forms and results in cytosolic nerolidol synthase and plastidial linalool synthase, consequently producing (*E*)-nerolidol and linalool *in planta*^33^. Another tea *TPS* recently reported is *CsNES*, which is only responsible for the formation of nerolidol, notably contributing to flavor and aroma of oolong tea^34^. However, a comprehensive study about tea *TPS* genes has not been reported yet.

Recently, genome databases from the tea cultivar “Yun-Kang 10” of *Camellia sinensis* var. *assamica* (CSA)^35^ and “Shu-Cha Zao” of *C. sinensis* var. *sinensis* (CSS)^36^ have been released. In the present study, eighty *CsTPS*-like genes were identified. Their phylogeny, structure, and expression patterns were comprehensively evaluated, with a special focus on their expression patterns under abiotic stress. Our findings provide a foundation for further exploration of tea *TPS* genes from other lineages with the aim of improving our understanding of the biosynthesis of terpenoids.

## Results

### Identification of *TPS* gene members in tea genomes

To retrieve tea *TPS* genes from recently publicized tea genome databases, the PF01397 and PF03936 domains, representing respectively N-terminal and C-terminal domains of TPS, were used. Those genes contained one or two domains of PF01397 and PF03936 were retrieved as tea *TPS* candidate genes from CSS and CSA genomes, meanwhile manual curation and validation of these *TPS* gene candidates were performed using each candidate genes as query to do BLASTP against the database at the National Center for Biotechnology Information (NCBI). A total of 80 and 60 *TPS*-like genes were found in CSS “Shu-Cha-Zao” (Supplementary Table S2**)** and CSA “Yunkang10” genomes (Supplementary Table S3), respectively. *TPS* genes from two tea genomes were found highly conserved in cDNA sequence with identity ranging from 77% to 100% but with varying sequence coverages. No *TPS* genes with identical sequences were found from the two genomes. Compared to the corresponding *TPS* homologues in CSS “Shu-Cha-Zao”, eight TPS genes from CSA “Yunkang 10” (highlighted in Supplementary Table S3**)** were found containing similar protein sequence length (90–110%), high identity at both cDNA and protein levels (>90%). However, 31 and 13 *TPS* genes respectively from CSA “Yunkang10” and “Shu-Cha-Zao” had incomplete protein sequences with less than 200 amino acid residues, all lacking either Pfam domain PF01397 or PF03936 (Supplementary Tables S2,S3). For further analysis, *TPS* gene models from the CSS genome assembly was employed.

**Figure 2:**
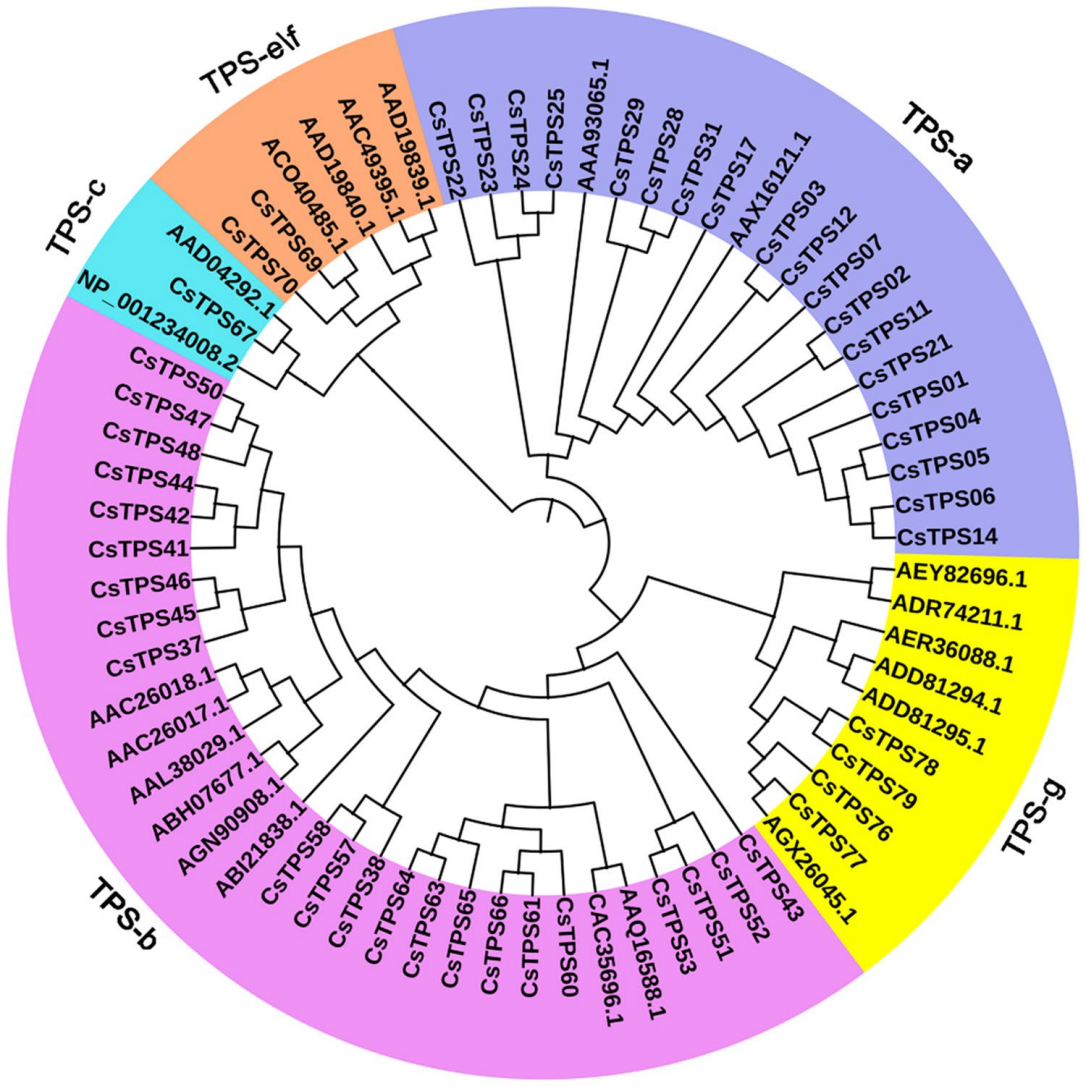
Phylogenetic analysis of tea *CsTPS* genes based on their predicted protein sequences. TPS, terpene synthases. The maximum likelihood algorithm tree was generated from an alignment of 70 TPS proteins, comprising 48 CsTPSs (the remaining *TPS* genes were too short or no common sites for meaningful alignment) and another 43 documented TPS from different plant species.

Phylogenetic analysis was performed using 48 *CsTPSs* (the remaining *CsTPS* genes were too short for meaningful alignment) and another 22 documented *TPS* from different plant species, indicating that tea *TPS* genes belongs to six subfamilies from TPS-a to TPS-g, but without TPS-d based on their protein sequences (Fig. 2). The TPS-a gene family in tea was the most expanded, with 36 genes, approximately 45% of the total *TPS* genes identified. This is in accordance with other plant species, including grape, *Arabidopsis*, and rice^24^. TPS-b gene subfamily as the second largest, included 30, about 37.5% of the total tea TPS genes. For the remaining *TPS* subgroups, only one gene encoding copalyl diphosphate synthase representing the TPS-*c* subgroup, eight representing the TPS-*e*/*f* subgroup, and five genes representing the TPS-g subgroups, respectively, were also identified (Supplementary Table S2). We designated these gene models of CSS genome as *TPS1* through *TPS80* according to the order of their subfamilies.

### Putatively functional *TPS* genes

For assessment of the tea *TPS* gene functions, full-length transcriptome sequencing data^37,38^ were employed to verify the putative functional *TPS* genes obtained from tea genome. Sequence similarity comparison between the full-length transcripts of *TPS* and genome assembly were conducted using BLAST with a threshold E-value < 1e-5 and identity >98%. After series of BLAST using the nucleotide sequence and manual evaluation, 80 tea *TPS* genes divided into three types: putative functional *TPS* genes with full coding sequences and complete structures (23 members); full-length coding *TPS* genes with disordered structure (9 members); and partial *TPS* genes (48 members), respectively (Supplementary Table S4). The first type of tea *TPS* genes had 23 members and all had an uncompromised open reading frame in either transcriptomic or genomic data (Table 1). Thus, they were most likely functional. *CsTPS57, CsTPS76*, and *CsTPS78* have been proved as active ocimene synthase, bifunctional linalool/nerolidol synthases and nerolidol synthase, respectively^33,34,39^. Another 20 *TPS* genes possessed full-length coding sequences either revealed by transcriptome data or genome assembly. These genes also possessed the intron-exon structure, i.e. seven exons for TPS-a, TPS-b, and TPS-g and 13 to 15 exons for TPS-e/f and TPS-c. Moreover, these genes contain the specific protein features of *TPS* gene family, such as the ‘DDXXD’ and ‘NSE/DTE’ motifs (Supplementary Table S4), which are important for metal dependent ionization of the prenyl diphosphate substrate in the C-terminal domain^23,40,41^. Out of these 20 *TPS* genes, 5, 11, and 2 were annotated as monoterpene, sesquiterpene and diterpene synthase genes in addition to 3 bifunctional *TPS* genes (mono- and sesqui-terpene synthase genes) (Table 1).

**Table 1:** Potential functional *TPS* genes in tea genome.

Name	Genome ID	Related transcript ID	Amino acid No.	Predicted functional
*CsTPS01*	TEA032539.1	CssPBTrans029702/PB.8683.1	567^a^	Germacrene D synthase
*CsTPS02*	TEA029356.1	CssPBTrans041118/PB.24310.30	537^a^	Germacrene D synthase
*CsTPS03*	TEA014184.1	CssPBTrans068400/PB.13101.1	569^a^	Germacrene D synthase
*CsTPS04*	TEA031969.1	PB.31351.2	572^c^	Germacrene D synthase
*CsTPS05*	TEA031966.1	PB.31351.8	522^c^	Germacrene D synthase
*CsTPS21*	TEA029348.1	CssPBTrans020115	567^c^	Germacrene D synthase
*CsTPS22*	TEA012463.1	PB.22660.1	547^a^	Germacrene D synthase
*CsTPS23*	TEA023168.1	CssPBTrans024950	540^a^	Germacrene D synthase
*CsTPS25*	TEA010551.1	CssPBTrans026737/PB.1889.1	552^c^	Germacrene D synthase
*CsTPS29*	TEA024081.1	None	541^a^	Germacrene D synthase
*CsTPS42*	TEA002963.1	CssPBTrans058925	568^a^	Myrcene synthase
*CsTPS43*	TEA014987.1		607^a^	Myrcene synthase
*CsTPS45*	TEA030379.1	CssPBTrans048789	575^c^	Myrcene synthase
*CsTPS47*	TEA022294.1	PB.21690.2	595^c^	Myrcene synthase
*CsTPS51*	TEA033306.1	PB.25018.1	528^a^	α-Farnesene synthase
*CsTPS57*	TEA004606.1	CssPBTrans046146/PB.522.1	585^b^	β-ocimene synthase
*CsTPS63*	TEA031457.1	PB.3574.1	549^c^	Tricyclene synthase
*CsTPS70*	TEA024176.1	CssPBTrans055745	781^c^	ent-kaurene synthase
*CsTPS73*	TEA019347.1		801^a^	(*E*,*E*)-geranyllinalool synthase
*CsTPS76*	TEA007191.1	PB.20489.1	575^b^	(3 *S*)-linalool/ (*E*)-nerolidol synthase
*CsTPS77*	TEA004822.1	PB.4391.1	557^a^	(3 *S*)-linalool/ (*E*)-nerolidol synthase
*CsTPS78*	TEA019472.1	PB.5304.3	546^b^	(3 *S*)-linalool/ (*E*)-nerolidol synthase
*CsTPS79*	TEA004657.1	PB.19331.4	598^a^	(3 *S*)-linalool/ (*E*)-nerolidol synthase

^a^The predicted protein depend on the genome assembly.

^b^These genes have been identified *in vivo* or vitro^13,33,34,39^.

^c^The predicted protein depend on the transcriptome data^37,38^.

The functions of the second type of 9 *TPS* genes were uncertain because full-length transcripts were not found or with low identity between the two sequence sources (the full-length transcriptomic data and genome assembly). In addition, their gene structures in genome assembly exhibited alterations in different extents compared to common structures of functional *TPS* genes, likely resulting in non-function or malfunction. For instance, *CsTPS24*, -37, -*41*, -*67* had one or more retrotransposon segment inserts, leading to disturbed gene structures. In particular, *CsTPS67* encoding a copalyl diphosphate synthase (CPS), harboring three retrotransposons with a total of 13,296 bp in length in the first intron (Supplementary Fig. S1), probably leading to failure of gene transcription. Even though it was unknown whether its transcription could occur regularly, Although the active domain ‘DXDD’ still present in its protein structure, 89 amino acids sequences in N-terminal is lost compared to *CPS* genes of other plant species identified. The *TPS* genes of this group were annotated as mono-, sesqui-, and di-terpene synthase genes (4, 4, and 2, respectively) according to manual BLASTP homologue searches.

The third type of tea *TPS* genes (48 members) with incomplete sequences contained no expected gene structures (i.e. intron-exon structure, open reading frames), compared to other plant species. The sequences of these *TPS* genes could not be validated using full transcriptomic data or RNA-seq data. Thus, these type of the *TPS* genes might not be functional, even though some of their incomplete sequences could be resulted from imperfect sequencing technique or transposon insertions (Supplementary Table S5**)**.

### Tempo-spatial expression patterns of *TPS* genes in different tea organs

**Figure 3:**
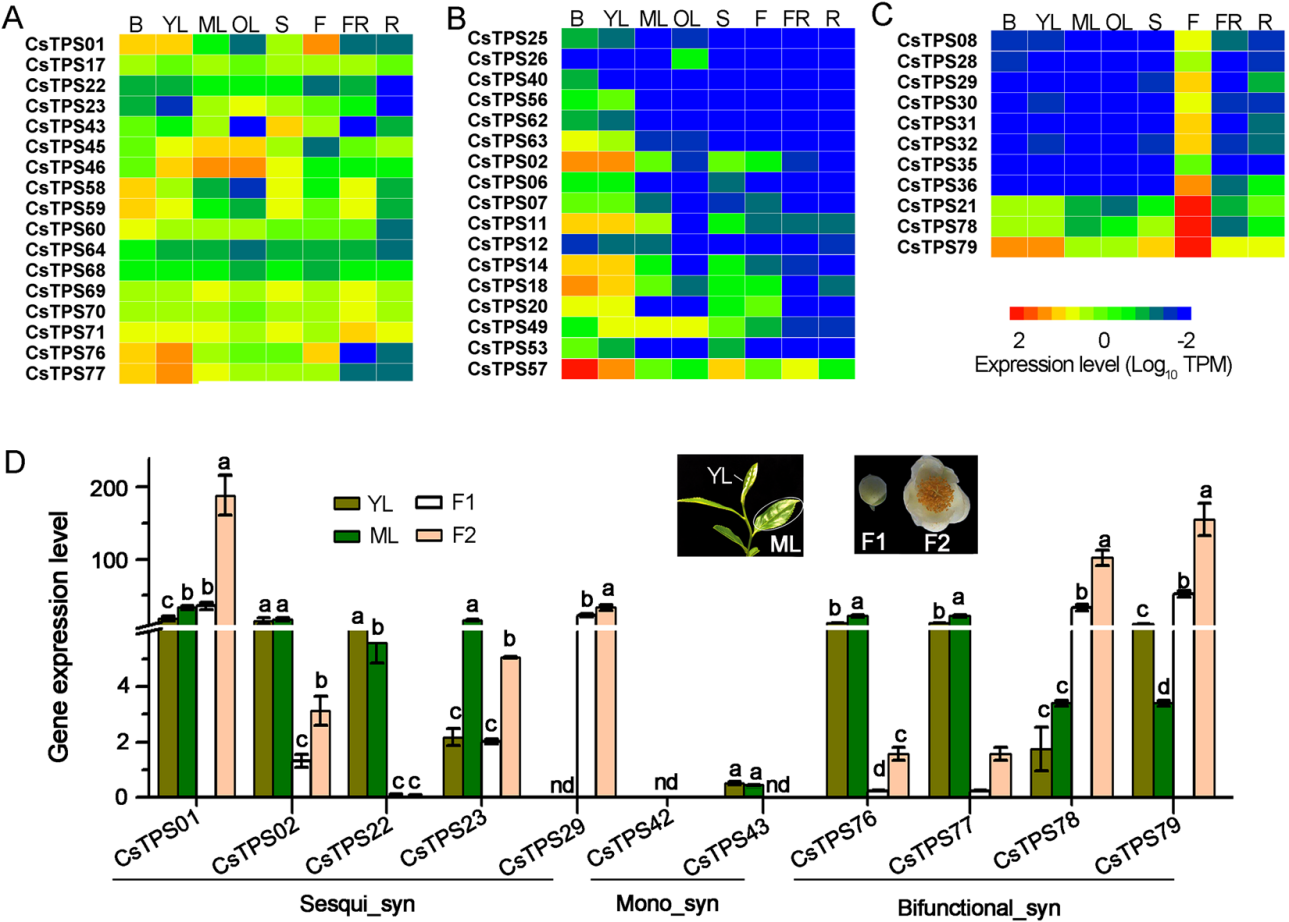
Tempo-spatial expression patterns of *CsTPS* genes in tea apical buds (B), young leaves (YL), mature leaves (ML), old leaves (OL), immature stems (S), flowers (FL), young fruits (FR) and roots (R). (**A**), *CsTPS* genes substantially expressed in eight tested organs with a few exceptions; (**B**), *CsTPS* genes predominantly expressed in leaves; (**C**), *CsTPS* genes primarily expressed in flowers; Transcripts per million (TPM) were used to evaluate gene expression levels; (**D**) qRT-PCR validation of transcriptomic data of the *CsTPS* genes in red in young and mature leaves (YL and ML) and flowers at the two developmental stages (F1 and F2) relative to the level of *CsTPS02* in F1. Mean levels (n ≥ 3) distinguished with different letters for each gene among different leaves and flowers are significantly different from each other (*p* < 0.05). Sesqui-syn, Mono-syn, and Bifunctional-syn represent sesquiterpenoid synthase, monoterpenoid synthase and bifunctional ternpenoid synthase, respectively.

To learn the tempo-spatial expression patterns of tea *TPS* genes, transcriptome datasets from eight organs of tea plants (i.e. apical buds, young leaves, mature leaves, old leaves, stem, flowers, fruit and roots) (tpia.teaplant.org) were employed (Supplementary Table S6). Logarithm value (Log_10_) of transcripts per million (TPM) of each annotated tea *TPS* transcript was obtained. Results indicated that *TPS* genes had distinct tempo-spatial expression patterns (Fig. 3). Seventeen genes, particularly *CsTPS17*, -*59*, -*69*, -*70*, and -*71*, were substantially expressed in all the tested organs with a few exceptions such as *CsTPS22*, -*23*, -*43*, -*58* and -*76* with undetectable levels in one or two organs (Fig. 3A**)**. On the contrary the majority of *TPS* genes that exhibited distinct tempo-spatial expression patterns. Six genes (*CsTPS25*, -*26*, -*40*, -*56*, -*62*, and -63) were solely and another 11 genes (*TPS02*, -*06*, -*07*, -*11*, -*12*, -*14*, -*18*, -*20*, -*49*, -*53*, and -*57*) were predominantly expressed in leaves (Fig. 3B**)**; another eight (*CsTPS08*, -*28* ~ -*32*, -*35*, and -*36*) and three (-*21*, -*78*, and -79) were almost exclusively and primarily expressed in flowers (Fig. 3C**)**. In addition, eight genes (*CsTPS09*, -*10*, -*13*, -*16*, -*27*, -*33*, -*65* and -*73*) had no detectable or trace transcript levels in all the tested organs, all of which belonged to the third group of putatively non-functional *TPS* genes except for *CsTPS73* (Supplementary Fig. S2 and Table S5).

Interestingly, 38 *TPS* genes expressed with substantial levels either in flowers or leaves were annotated as sesquiterpene synthases genes, while monoterpenoid synthase genes largely with low transcription levels were only seven (*CsTPS40*, -*43*, -*45*, -*46*, -*49*, -*60*, -*62*, and -*63*). These mono-*TPS* genes were restricted to be expressed in vegetative organs except for *CsTPS43*, -*46* and -*49* which also expressed in flowers.

qRT-PCR was performed to validate the expression patterns of *CsTPS* genes using some of putatively functional genes in Table 1. Significantly distinct transcript levels of *CsTPS01*, and -*2* were found in young leaves (YL) compared to the corresponding levels in flowers using qPCR methods. Higher transcript levels of *CsTPS22* and -*23* were found in mature leaves (ML) than in both flower buds (F1) and open flowers (F2). Similarly, higher transcript levels of *CsTPS43*, -*76*, and -*77* were also found in leaves than in flowers. However, for *CsTPS29*, -*78*, and -*79*, higher transcript levels were noted in flowers compared to those in leaves (Fig. 3D). It is interesting to note that *CsTPS29* was exclusively expressed in flowers. All these quantitative analysis results obtained using qRT-PCR were well consistent with the transcriptomic data (Fig. 3A–C). Gene annotation indicates that *CsTPS01* and -*02* likely encoded sesquiterpene synthases and that *CsTPS76*-*79* were annotated as difunctional *CsTPS* genes, among which *CsTPS76* has been proved as linalool synthase and nerolidol synthase^33^.

### Terpenoid profiling in tea leaves and flowers

**Figure 4:**
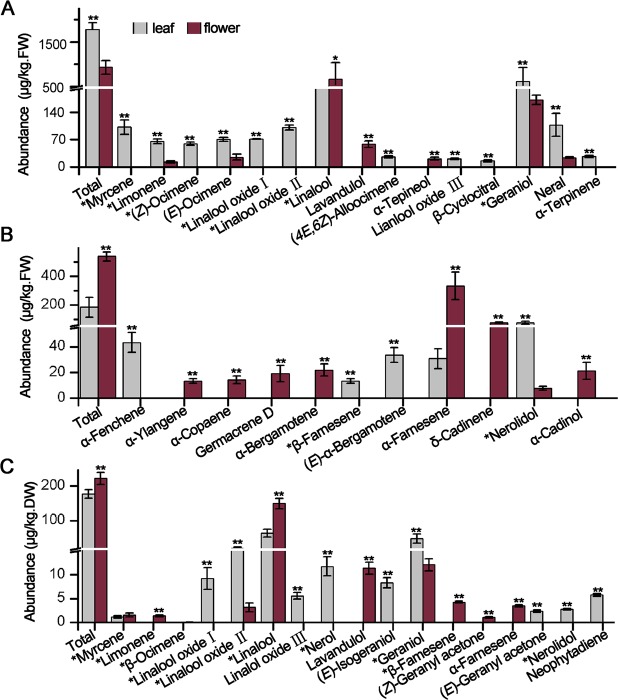
The abundances of terpenoids in tea leaves and flowers. (**A**), internal monoterpenes; (**B**), internal sesquiterpenes; (**C**), emitted terpenoid compounds. Significant differences between leaves and flowers are indicated (**p ≤ 0.01, *p ≤ 0.05). All data are expressed as mean ± S.D. (n = 3). The compounds labeled with stars were identified and quantified with authentic standards and standard curves, respectively.

It was obvious that transcription regulation is crucial for terpenoid production in plants^7,22^. Intact tea leaves usually do not release any perceivable volatiles, but they contain significant amount of terpenoid volatile precursors, which contribute to the aroma formation of made teas^11^. On the contrary, tea flowers have a great potential to be utilized because of their abundant functional molecules such as saponins, polysaccharides, aromatic compounds and functional proteins^42^ and emit substantial volatiles, including terpenoid compounds^16^. Therefore, terpenoid profiling in tea leaves and flowers combined with *CsTPS* transcriptomic analysis may reveal the association between *CsTPS* gene expression and their chemical products, which could be helpful for further *CsTPS* gene functional characterization. In the present study, the abundance of the internal volatile terpenoids and emitted volatile terpenoids (collectively from free and hydrolyzed glycosides) from tea leaves and flowers were analyzed by GC-MS. In total, seventeen monoterpenes and eleven sesquiterpene compounds were identified from tea leaves and flowers (Fig. 4A,B). The total abundance of all monoterpenes extracted from flowers was lower than that from leaves and almost all the monoterpenes detected from leaves were more than their counterparts in flowers, except for linalool, lavandulol and α-terpineol.

Linalool and its oxides were the most abundant among all the monoterpenoids, followed by geraniol, two accounting for 66% and 76% of the total monoterpenoid amounts in tea leaves and flowers, respectively (Fig. 4A). However, the total abundances of internal sesquiterpenoids and emitted terpenoids in flowers were higher than those in leaves (*p* < 0.01) (Fig. 4B,C). α-Farnesene was the most abundant sesquiterpene detected from flowers, contributing to approximately 70% of the total amount of sesquiterpenoids in tea flowers, while nerolidol is the most abundant sesquiterpene in leaves, contributing to 38% of total sesquiterpenoids abundance. *CsTPS51*-*56*, were annotated as putative α-farnesene synthase according to BLASTP results (Supplementary Table S2). Nevertheless, their transcription only found in leaves (Supplementary Fig. S2), indicated that abundant emission of α-farnesene in flower might be depended on other *TPS* genes, such as *CsTPS28*-*32*. Another possibility could not be excluded that the function of these *CsTPS* genes could be wrong. Furthermore, all sesquiterpenoids were mainly generated from flowers, rather than leaves, except for nerolidol (Fig. 4B). As above the gene expression patterns analysis, sesquiterpenoids were abundant in flowers than leaves mainly attributed to the higher accumulation of their transcripts in tea flowers.

On the contrary, compared to 17 and 11 sesqui- and mono-terpenoids respectively detected, reduced numbers of emitted monoterpenoids (11) and sesquiterpenoids (2) in addition to a diterpene (neophytadiene) were detected from leaves and flowers, probably attributing to some terpenoids that is not readily emitted or unstable (Fig. 4C). It is interesting to note that emitted linalool from homogenized flowers was significantly higher than that from homogenized leaves (*p < *0.01) while the opposite was found for geraniol. Either organic extraction analysis or emitted terpenoids analysis, linalool and geraniol were no doubt as the most abundant monoterpenoids in tea plants. It was clear that *CsTPS77-79* are responsible for the linalool biosynthesis. However, the key enzyme involved in geraniol biosynthesis in tea plants is still unknown.

### *TPS* gene expression under abiotic stress conditions

Tea plants are often subjected to drought, salinity, cold and other stress conditions most likely they are generally grown in mountainous regions with shallow soils, thereby possibly affecting the production of terpenoids as responding to stress conditions^7,22,33^. Hence, transcriptome data were employed to investigate the expression alterations of tea *TPS* genes responding to the treatments of stress or stress signal molecule methyl jasmonate (MeJA).

**Figure 5:**
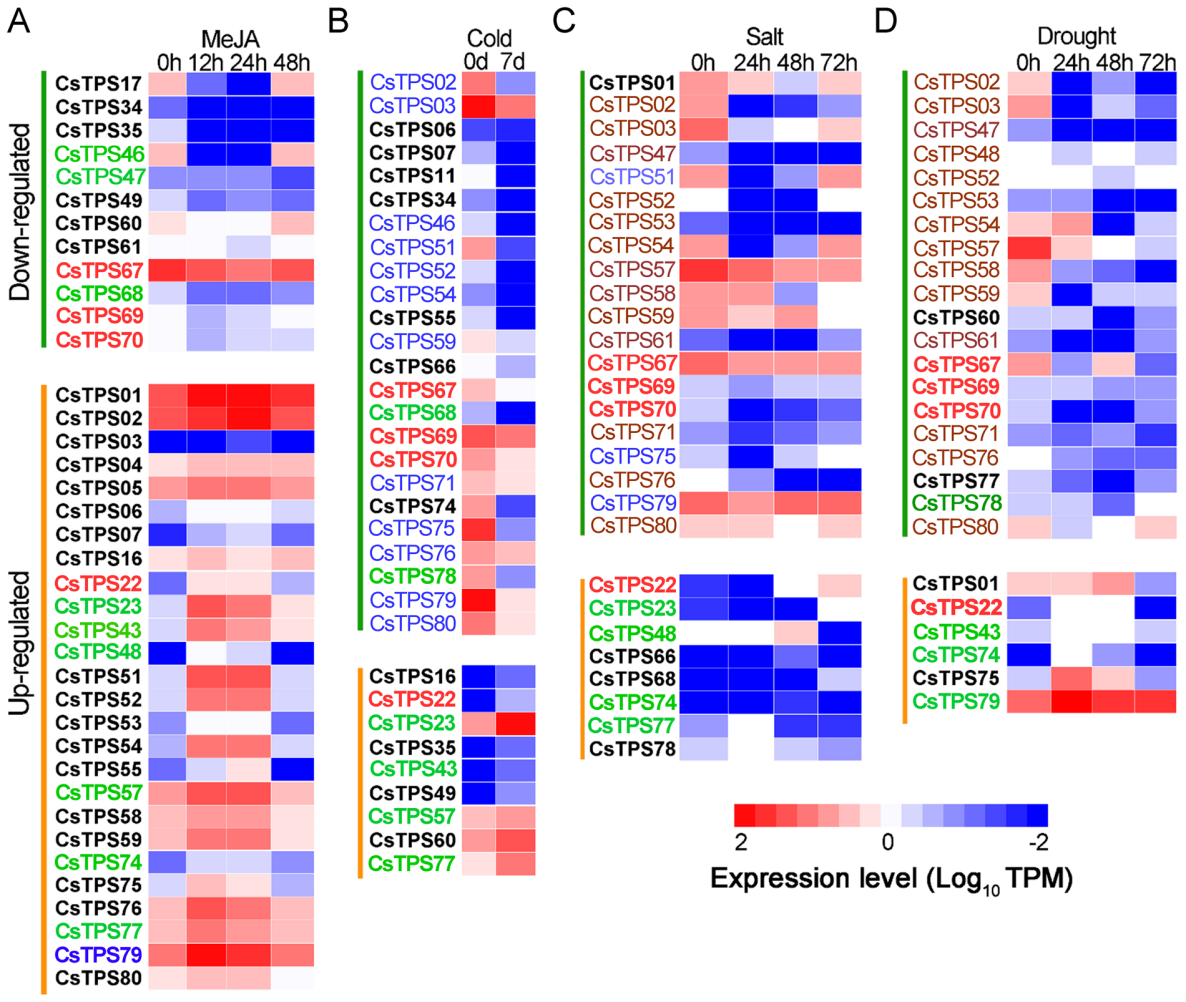
Transcriptomic responses of al the 80 *CsTPS* genes under different abiotic stress conditions. (**A**), Treated with 1 mM MeJA; (**B**), treated with cold acclimation (10 °C-4 °C for 7 d); (**C**) Treated with 200 mM NaCl; (**D**) treated with 25% PEG. Transcripts per million (TPM) were used to evaluate gene expression level. Genes in red and bold were either enhanced or suppressed across all the treatments; Genes in dark red were suppressed in both salt and PEG-induced drought treatments. Genes in green shared the same changes among two or three treatments either enhanced or suppressed.

MeJA treatment differentially regulated *CsTPS* gene expression (Fig. 5). Compared to nontreated control, 12 hr after MeJA treatment, 12 *CsTPS* genes were suppressed in the range from 1.3- to 542.3-fold, the highest for *CsTPS46* and the lowest for *CsTPS61*, with the average suppression of 59.0-fold (Fig. 5A). The suppression of gene expression was alleviated for 11 *CsTPS* genes 24 hr after the treatment, except for *CsTPS17*, whose expression was further suppressed from 45.0-fold (12 hr after the treatment) to 457.7-fold (24 hr after the treatment). The majority of the suppressed genes were recovered 48 hr after the treatment. On the contrary, 12 hr after the treatment 27 *CsTPS* genes were enhanced in the range from 1.2- to 94.2-fold, the highest for *CsTPS48* and the lowest for *CsTPS78*, with the average enhancement of 13.2-fold (Fig. 5A). Time course study indicated enhanced expression of many *CsTPS* genes declined 24 hr after the treatment, except for *CsTPS03*, -*07*, -*51*, -*52*, -*54*, and -*55*, whose expression levels were further enhanced. Whereas the MeJA enhanced expression for almost all the *CsTPS* genes was recovered back to non-treated control levels 48 hr after the treatment.

Under cold acclimation (between 10 °C to 4 °C) for 7 days, more *CsTPS* genes (24) were suppressed than the number of enhanced genes (9) (Fig. 5B). compared to the non-acclimated controls, transcript suppression ranged from 1.4- to 32.3-fold with an average suppression 11.8-fold. The most severely suppressed was *CsTPS78*, followed by *CsTPS11*(28.4-fold) and *CsTPS46* (26.4-fold). On the contrary, expression enhancement for another 9 *CsTPS* genes ranged from 1.3- to 37.8-fold with the average of 12.6-fold. The maximal enhancement occurred to *CsTPS43*, followed by *CsTPS35* (23-fold) and *CsTPS16* (19.5-fold) (Fig. 5B).

Salinity stress primarily led to varying expression reduction of 20 *CsTPS* genes in the range from 1.4- to 47.6-fold 24 hr after the treatment. The most severely suppression was found for *CsTPS02*, followed by *CsTPS54* (43.6-fold) and *CsTPS51*(36.2-fold). Time-course study revealed that the suppression was alleviated as the time proceeded after the treatment. Eight *CsTPS* genes were slightly salt stress induced. The most dramatically enhanced was *CsTPS22* (11.6-fold), followed by *CsTPS68* (9.2-fold), all occurred 72 hr after salinity treatment (Fig. 5C).

Furthermore, PEG treatment for a certain period to simulate drought treatment^41^ also resulted in the suppression of 20 *TPS* genes in the range from 1.3- to 324.7-fold, with the average of 36.8-fold. The most dramatically suppressed gene expression occurred to *CsTPS03*, followed by *CsTPS59* (151.2-fold) and *CsTPS02* (142.9-fold). Six genes were PEG enhanced with the most notably enhancement occurred to *CsTPS74* (48.3-fold), followed by *CsTPS75* (17.9-fold) 24 hr after the treatment (Fig. 5D).

It was interesting to note that across all the four different treatments three genes (*CsTPS67*, -*69*, and -*70* in red and bold) were all suppressed while *CsTPS22* (in red and bold) was enhanced (Fig. 5). Moreover, out of 20 *CsTPS* genes suppressed by salinity, 17 were also suppressed by PEG-induced drought (in dark red) and 15 were suppressed by cold acclimation (in blue). In addition, some genes (in green) shared the same suppression or induction alteration among two or three different treatments, suggesting that some genes responded to the abiotic stresses in the similar ways.

**Figure 6:**
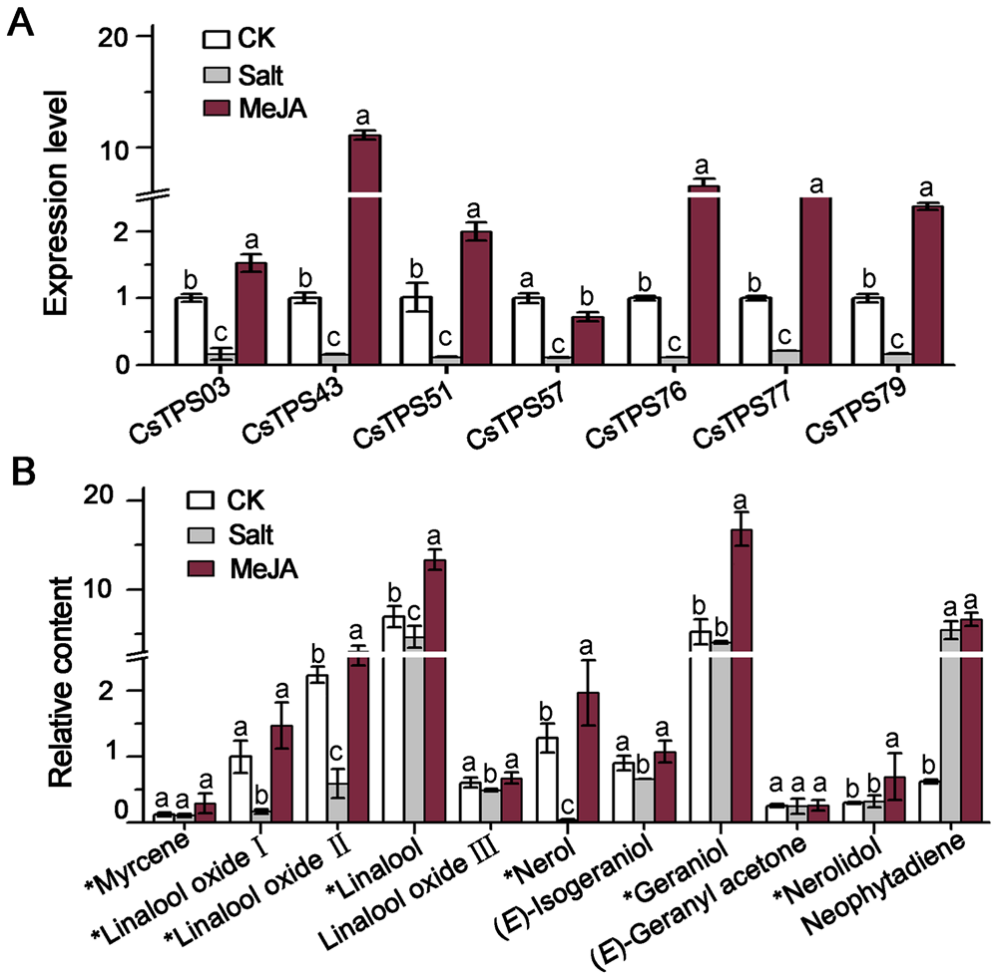
Expression pattern of *TPS* genes, and terpenoids production under different abiotic stresses. (**A**), gene expression patterns validated by qRT-PCR; (**B**), Stress induced changes in the abundance of emitted terpenoids. Means distinguished with different letters among non-treated control, MeJA and salt treatments are significantly different from each other (*p* < 0.05). All data are expressed as mean ± S. D. (n ≥ 3). The compounds labeled with stars were identified and quantified with authentic standards and standard curves, respectively.

Further validation of the transcriptomic analysis result was conducted using qRT-PCR approach with some *CsTPS* genes and GS-MS quantification of volatile terpenoid abundance in tea leaves treated with MeJA or salinity (Fig. 6). Our qRT-PCR results confirmed the salinity suppression and MeJA induction of *CsTPS* genes (*CsTPS03*, -*43*, -*51*, -57, -*76*, -*77*, and -*79*) (*p* < 0.05), well consistent with transcriptomic data (Fig. 6A). Moreover, salinity stress resulted in a significant abundance reduction in linalool and its three oxides and nerol (*p* < 0.05). Geraniol was slightly decreased (*p* > 0.05) due to salinity treatment. Nevertheless, an increase (*p* < 0.05) in linalool, nerol, geraniol, and nerolidol was induced by MeJA (Fig. 6B). Neophytadiene, a putatively identified diterpene volatile, was also increased by both MeJA and salinity treatments. It was interesting to note that the amounts of linalool, geraniol and nerolidol in leaves were increased by 192%, 318% and 232%, respectively (Fig. 6B).

## Discussion

*C. sinensis* is an important economic crop widely grown in mountainous regions in South-Eastern Asian countries. Such a type of the geographic plantation often turns the tea plants under various and severe stress conditions such as drought, cold damage, herbivore attack. Tea plant volatile terpenoids play significant roles not only in resistance against stress conditions^12,13^, but also in tea beverage flavor formation^11,14,15^. Studies on tea plant *TPS* family genes and their responses to different stress conditions, are crucial for tea plant productivity and tea flavor improvement.

Plant *TPS* gene families are a medium sized group, although the numbers of *TPS* families from different plant species vary significantly^24^. For instance, in *Arabidopsis* 40 genomic *AtTPS* genes are found, but 32 are functional^42^; In grape (*Vitis vinifera*), there are 152 *TPS*-like genes and 62 are functional^23^; In tomato (*Solanum lycopersicum*) out of 44 *TPS*s, 29 are functional or potentially functional^28^. In apple, only 10 *TPS* genes out of 55 are functional^25^. In this study, different numbers of *TPS* genes were found in the recently released genomes of CSS “Shu-Cha-Zao” (80 genes) and CSA “Yunkang10” (60 genes), probably because of imperfect sequencing technology and genome assembly. Although the sequences of many *TPS* genes from both genomes were incomplete, similarity of orthologous *TPS* genes at nucleotide level between the two genomes was as high as 96%, slightly higher than 92%, the average similarity of orthologous genes at DNA levels between the two cultivars^36^, suggesting that the orthologous TPS genes likely had similar function in the two different cultivars, but with some distinct variations.

Among 80 *CsTPS* genes found in “ Shu-Cha-Zao” genome, three have been proved functional in previously published reports^33,34,39^ and 20 others were most likely functional due to their possession of expected full-length coding sequences and gene structures based on genome assemblies or/and full-length transcriptomic data. In addition to the three functional *TPS* genes, five, eleven and two were annotated as mono-, sesqui-, di-terpene synthase genes. The same function annotation of multiple *TPS* genes in the public databases was noted, probably because the functions of some genes were obtained from *in vitro* assays only. For examples, some terpene synthases were found bifunctional *in vitro* to produce both linalool and nerolidol, but actually monofunctional in planta^43,44^. It has been indicated in many cases that the function obtained *in vitro* are different from actual function in planta^33^.

For the remaining 57 *CsTPS* genes, their functions were uncertain due to their incomplete sequences or untypical gene structures obtained from the currently available genome databases, which requires improvement in its sequence precision. Full sequences of these genes should be obtained for their functional validation *in vitro* and in plants. Additionally, the possibility also could not be excluded that low number of predicted functional tea *TPS* genes perhaps attributes to gene duplication and simultaneous generation of many meaningless sequences in genome occurred over tea plant evolution^36^. Transposable elements (TEs), are the chief mechanistic drivers of genome evolution, representing at least 64% of the assembly (excluding undefined base Ns)^36^. It was found that 19 of tea *TPS* genes containing one or more transposable elements (TEs) within intron (Supplementary Table S5), likely leading to neofunctionalization of tea TPS genes. Further functional characterization is required to reveal their functions specifically in tea plants. In addition, transcriptomic data applied in this study from different groups indicated that *CsTPS13*, -*27*, -*33*, and -*65* genes had undetectable transcript levels in all different tea organs nor under all the four tested stress treatments (Supplementary Fig S2 and S3), possibly suggesting that they might be silenced genes.

In the present study, tempo-spatial expression patterns of *CsTPS* genes was noted and validated using qPCR approach. Interestingly, *CsTPS* genes with high transcript levels in both flowers and leaves were all sesquiterpene synthase genes. It was noted that 17 sesquiterpene synthase genes were higher expressed in flowers than in leaves while limited monoterpene synthase genes maintained substantial transcript levels in tea leaves or any other tested organs, suggesting a strict regulation of terpenoid production. Moreover, transcriptomic data indicated that distinct tempo-spatial expression patterns of *CsTPS* genes could be significantly affected when the plants were subjected to different stress conditions, which were confirmed with qPCR data and volatile profiling results in this study. Many *CsTPS* genes such as *CsTPS23*, -*25*, -*43*, -*51*, -*52*, and -*76* were significantly induced by MeJA treatment whereas the majority of tea TPS genes were suppressed by salinity, drought, and coldness. *CsTPS76* is known being MeJA induced^33^ due to the presence of G-boxes in its promoter, which can interacts with MYC2 transcription factor of JA signalling pathway^45^ to deal with many stress conditions^46^. It is assumed that many of those MeJA induced *CsTPS* genes might contain *cis*-elements able to interact with JA signalling pathway. Our leaf chemical profiling data revealed a significant MeJA enhancement of multiple monoterpenoid volatiles such as linalool, geraniol and their derivatives as well as sesquiterpenoid nerolidol in tea leaves. Reduced production of many of the above-mentioned compounds in the leaves subjected to salinity were also noted. These chemical profiling results were well consistent with the transcription quantification data. Although many TPS genes, including those encoding mono- and sesqui-terpenoid synthases, were MeJA enhanced or salt suppressed, significant abundance changes were mainly detected in very limited numbers of the monoterpenoid compounds such as linalool, geraniol and their derivatives, rather than sesquiterpenoids except nerolidol. These were probably because of the following reasons: 1) detected terpenoids such as linalool and geraniol are likely abundant in fresh tea leaves^15^; 2) preference of SPME volatile collection to those small terpenoid molecules with high volatility. Moreover, it was noted that three *CsTPS* genes (-67, -69 and -70) were suppressed by all the four treatments and many more were suppressed by both salinity and PEG-induced drought, even by cold treatments. It is known that *TPS* genes are significantly involved in the resistance mechanism of plants under different abiotic stresses, thereby regulating terpenoid metabolism^47,48^. In addition, many stress conditions can activate the same signalling pathways in plants such as “reactive oxygen species”, “Mitogen-Activated Protein Kinase (MAPK) cascades”, “ABA/JA/Ethylene signalling pathways”^49^.

Previous study indicates that tea leaves contain abundant and diverse monoterpenoids but emit none of them unless damaged, while tea flowers possess sesquiterpenes and emit both sesquiterpene and monoterpenes^33^. In this study, significantly more abundant sesquiterpenoids were detected in flowers than in leaves and a quite number of monoterpenoid volatiles were detected from leaves, indicating distinct patterns of volatile terpenoid production and emission in tea plants, well consistent with distinct *CsTPS* expression patterns between leaves and flowers. This is also in agreement with the findings obtained in Arabidopsis vegetative and reproductive organs^3,50^. In this study, the finding that all the monoterpenes in leaves were more than their counterparts in flowers with a few exceptions was consistent with the high transcript levels of some monoterpene synthases in leaves, such as *CsTPS37*-*50* and *CsTPS 60*-*66* (Supplementary Fig. S2). Although a few monoterpene synthase genes maintained substantial transcript levels in tea leaves, diverse monoterpenoid volatiles were detected in tea leaves. This is not surprising because a terpenoid synthase usually can catalyze a single molecule into different products^18,50^. In this study, both leaves and flowers can release many volatile terpenoids, particularly linalool and geraniol, after tissue homogenization. This is partly because these compounds were present as internal free form, but also because they are accumulated as glycosidically bound forms^11^. Ohgami *et al*.^19^ demonstrated that geraniol and other volatiles produced in tea leaves can be sequentially glycosylated into β-primeverosides by two glycosyltransferases so that they can be stored in leaf tissues. For a better understanding the mechanisms of tea terpenoid production in tempo-spatial patterns, more efforts should be made to combine the studies with *CsTPS* gene expression manipulation with those on terpenoid profiling.

## Materials and Methods

### Plant materials and stress treatments

Six-year old plants of *C. sinensis* var. *sinensis* (CSS) cv. “Shu-Cha-Zao” grown at the experimental farm of Anhui Agricultural University in Hefei, China were used in this study. Leaves and flowers at different developmental stages were excised for gene expression and terpenoid analysis. For salinity and methyl jasmonate (MeJA) treatments, 12 one-year old potted plants of the same cultivar were placed in the green house (at 26 °C/22 °C, day/night and a 16 h photoperiod at 100 μmol photons m^−2^ s^−1^). For the salinity treatment, the same potted plants (12) were irrigated each with a 150 mM NaCl solution until it dripped out from the pot bottom and then the plants were left un-watered for the next 5 days. The control plants were irrigated in same way using tap water. Then leaves from the salt treatment were collected over a 72 h period and immediately frozen in liquid nitrogen and kept in −80 °C for future use. For the MeJA treatment, plants were sprayed with 1 mM MeJA in 0.05% DMSO until the solution dripped off the sprayed leaves. Control plants were sprayed with the same solution without MeJA. Leaves were collected up to 48 hours after the MeJA treatment, and then frozen immediately in liquid nitrogen and stored at −80 °C for further study.

### Identification of tea *TPS* family members

The recently released tea genome assemblies of *C. sinensis* var. *assamica* (CSA) cv. “Yunkang10” and *C*. *sinensis* var. *sinensis* (CSS) cv. “Shu-Cha-Zao”^35,36^ were used in this study. Full length transcriptomic databases and RNA-seq datasets were retrieved from the Tea Plant Information Archive (http://tpia.teaplant.org/)^37,38^. PF01397 and PF03936 domain data which represent respectively the N-terminal and C-terminal domains of TPS from the Pfam database (http://pfam.xfam.org/)^51^ were used to identify the members of the tea *TPS* gene family. Manual annotation was also conducted based on the results of a BlastP against *TPS* genes from the GenBank of The National Center for Biotechnology Information (www.ncbi.nlm.nih.gov) and Swiss-Prot (www.uniprot.org/blast/).

### Phylogenetic analysis

Multiple sequence alignments of TPS protein sequences in tea and their homologues from other plant species were conducted using ClustalX in MEGA 5.0 using default sets. The alignment was conducted using 70 TPS proteins, comprising 48 CsTPS and 43 documented TPS from different plant species (the remaining *CsTPS* genes were too short or did not possess any common sites for meaningful alignment). The obtained alignment was used as the input for the maximum likelihood algorithm in MEGA5.0 software to construct phylogenetic trees. Subfamilies are divided based on cluster analysis^52^.

### Gene expression analysis

For gene expression analysis, transcriptomic data and their validation using quantitative real time polymerase chain reaction (qRT-PCR) were performed. In order to characterize tempo-spatial gene expression patterns in the tea plant, transcriptomic data from apical buds (tightly folded young leaves), young leaves (the first or second unfolded leaves of growing shoots), mature leaves (the fourth leaf with dark green color), old leaves (the leaves at shoot base), immature and unlignified stems, flowers, young fruits and tender roots and of tea plant under diverse biotic and abiotic stresses were retrieved from the Tea Plant Information Archive (TPIA) database^37^. An average of 11.8 Gb of clean RNA-seq data were generated electronically for each of the eight tissue types from published data^37^. For the treatments of salinity (200 mM NaCl) and drought (induced by 25% PEG, Polyethylene glycol), equal amounts of total RNA extracted from tea shoot tips collected at each time interval (0 h, 24 h, 48 h, 72 h) after stress or non-stressed treatment were pooled together for transcriptome analysis^53^. To study the effects of cold acclimation (10 °C ~ 4 °C for 7 days) and non-acclimation (25~20 °C, CK) on tea plant *TPS* gene expression, data were retrieved from TPIA database^37^. For MeJA treatments, publicized RNA-seq data resulted from shoot-tips (including folded and the first two unfolded young leaves) excised at four different time intervals (0, 12, 24, 28 hr) from 2000 tea plants after evenly sprayed with 1.1 mM MeJA solution^54^. Transcripts per million (TPM) of RNA molecules were used to evaluate expression level.

For qRT-PCR validation of the transcriptomic analysis results, total RNA was extracted from leaf and flower samples either at different developmental stages, or from the potted plants treated with NaCl or MeJA in this study using the RNA prep pure Plant Kit (TianGen Biotech., Ltd, Beijing, China). cDNA was synthesized using a PrimeScriptTM RT reagent kit with gDNA Eraser (TaKaRa, Tokyo, Japan). Quantification of tea *TPS* genes, which are most likely functional, was performed using gene specific primers (Supplementary Table S1) and the 18srRNA as an internal reference. qRT-PCR was performed using the BioRad CFX96 real-time PCR system as applied before^33^. The relative expression level of tea *TPS* genes was calculated using the 2^−ΔΔCT^ method^55^. Each quantification had three biological replicates.

### Collection of terpenoid volatiles

Organic solvent extractions were used to collect the internal volatiles that were not readily emitted but were stored in the tea samples according to Zeng *et al*.^56^. For extraction of terpenoid compounds in tea leaves or flowers, plant samples were homogenized in liquid nitrogen, and then a mixed pentane/ethanol solvent (1:1, v/v) was applied to the homogenate, followed by centrifugation at 10000 *g* for 10 min. One μl of 0.1% ethyl decanoate was added into the supernatant as an internal standard. Liquid samples were analyzed by gas chromatograph-mass spectroscopy (GC-MS) according to Liu *et al*.^33^. The solid phase microextraction (SPME) method was used to collect the released volatiles in the headspace of homogenized plant samples according to a previous report^56^. Collected volatiles were resulted from the release of free and glycosidically bound volatiles after hydrolysis. Tea samples collected from leaves and flowers and different abiotic stress treatments were freeze-dried under vacuum at −58 °C. The dried sample (0.2 g) was then placed into a glass sampling vial (20 mL) with the addition of 5 mL boiled water, then volatiles were collected using a solid phase microextraction (SPME) fiber (65 μm PDMS/DVB, Sigma-Aldrich, Shanghai, China) after placement in a 70 °C water-bath for 30 min. Ethyl decanoate (0.01%) was added to the samples as the internal standard. All of the volatile compounds absorbed onto the SPME fiber were desorbed in the GC-MS injector at 250 °C for 5 min and then immediately analyzed by GC-MS.

### GC-MS analysis

To identify the volatile compounds, a gas chromatograph (Agilent 7697 A) and as mass spectrometer (Agilent 7890 A) outfitted with a DB-5 capillary column (30 m × 0.25 mm × 0.25μm; Agilent) were used in this study. Helium was used as the carrier gas. The initial GC oven temperature was at 50 °C for 1 min and increased to 100 °C at a rate of 10 °C/min (held for 1 min); then ramped up to 200 °C at a rate of 4 °C/min (held for 1 min), and then increased to 280 °C at 16 °C/min (held for 7 min). Chemicals were identified by comparing the retention time and mass spectrum either with those of authentic standards or the NIST database. Compounds were quantified based on calibration curves established using a series of diluted solutions prepared with authentic standards^15^ or based on the peak areas of the internal standard as relative content.

### Statistical analysis

The SPSS statistical package (Version 19.0) was used to conduct one-way analysis of variance (ANOVA) among different samples over three replications. A Least-significant difference (LSD) test and Bonferroni test were applied to determine the significance of differences (p < 0.05, p < 0.01).

## Supplementary information

Supplementary Information.
