# Supplementary material for: Analysis of Terpene Synthase Family Genes in Camellia sinensis with an Emphasis on Abiotic Stress Conditions
Source: Sci Rep. 2020 Jan 22;10:933. doi: 10.1038/s41598-020-57805-1 (PMC6976640; doi:10.1038/s41598-020-57805-1)
Supplement: Supplementary file 1 — Supplementary Information. [file 41598_2020_57805_MOESM1_ESM.pdf]

Analysis of Terpene Synthase Family Genes in *Camellia sinensis* with an Emphasis on Abiotic Stress Conditions

Han-Chen Zhou[1,2#,] Lubobi Ferdinand Shamala[1#], Xing-Kai Yi[1,3], Zhen Yan[1], Shu Wei[1*]

[1]State Key Laboratory of Tea Plant Biology and Utilization, Anhui Agricultural University, Hefei, China

[2]Tea Research Institute, Anhui Academy of Agricultural Sciences, Huangshan, China

[3]Horticultural Institute, Anhui Academy of Agricultural Sciences, Hefei, China

*** Correspondence:**
Shu Wei
weishu@ahau.edu.cn

Supplementary Information

Supplementary Table S1 Primer for tea TPS genes analysis

Gene Name	Primer for qRT-PCR
CsTPS01	F:CATCCTACCATTTGGGGAGATCATT<br>R:GCTGATCATTAGCTGCCACTAGCAT
CsTPS02	F:TCACTCGACGCTCAGCCAATTATCA<br>R:TGGATTGCATCGATGAAGTTCAGTT
CsTPS03	F:CACATCTCAGGATGCGAGGAGAAGA<br>R:AATGGATGGGCTGATTCAGAGCATG
CsTPS22	F:AGGAATGCCCAGATTAGAGGCCAGG<br>R:TGGTGCAACCTTTGTAAAAGATTGA
CsTPS23	F:TGAGGTGAGAGGGATGCTACGTGAT<br>R:TCATCATCATCACCACCACCACCAC
CsTPS29	F:CCTACTCGCCACTTGGCCAAATTTC<br>R:CGGCTACCACCATGCTCCTCACTTG
CsTPS42	F:TAGAGAAGCTGGAGGAGCCATTGGA<br>R:GCGCCAATTCATTTTGTGATCAATA
CsTPS43	F:TTCAAGTGAATCCACGACGATCAGC<br>R:ATTCAACTCTTCTAATTTGGTCCCA
CsTPS51	F:CTTTAGGCTCCTCAGGCAGCATGGA<br>R:AGGCTTCAAGAAGCTCAAGCATTGC
CsTPS73	F:TTTCCCCATCTGCCTATGACACTGC<br>R:ACCATCATTGCCAACTTCACCCCAA
CsTPS76	F:TAACGGTTTGATGCCACATCTTTCT<br>R:CCGTGTCTTTCCACCACTTTGATAC
CsTPS77	F:GCAAGAAGGGTACAATGTGCCCGCG<br>R:CTGCGGCTTGATCAAGAATGTCTTC
CsTPS78	F:AGGCATTGATTACCACTTCCGGGAG<br>R:GCACATTGTAGCCTTGTTGCCTCAA
CsTPS79	F:CATGATCGATGCCCTCCAACG<br>R:GTTGCCTCAACAGACGAAAGCGA

Supplementary Table S2.   Identification of tea TPS gene family in CSS genome

NO.	Name	Gene ID	Location	Protein(aa)	Nucletide(bp)	Exon	Pfam analysis	SWISS PROT Annotation	TPS sub-familly
**1**	CsTPS01	TEA032539.1	Scaffold2014:1796883:1802367:-	567	1704	7	PF01397 / PF03936	TPSGD_VITVI, (-)-germacrene D synthase	TPS-a
**2**	CsTPS02	TEA029356.1	Scaffold559:448741:453375:+	537	1614	7	PF01397 / PF03936	TPSGD_VITVI, (-)-germacrene D synthase	TPS-a
**3**	CsTPS03	TEA014184.1	Scaffold2694:466713:471430:-	569	1710	7	PF01397 / PF03936	TPSGD_VITVI, (-)-germacrene D synthase	TPS-a
**4**	CsTPS04	TEA031969.1	Scaffold982:939616:948654:+	1042	3129	8	PF01397 / PF03936	TPSGD_VITVI, (-)-germacrene D synthase	TPS-a
**5**	CsTPS05	TEA031966.1	Scaffold982:979330:981825:+	441	1326	5	PF01397 / PF03936	TPSGD_VITVI, (-)-germacrene D synthase	TPS-a
**6**	CsTPS06	TEA025177.1	Scaffold7725:83168:89037:+	624	1875	9	PF01397 / PF03936	TPSGD_VITVI, (-)-germacrene D synthase	TPS-a
**7**	CsTPS07	TEA010895.1	Scaffold1955:1632511:1641345:+	559	1680	8	PF01397 / PF03936	TPSGD_VITVI, (-)-germacrene D synthase	TPS-a
**8**	CsTPS08	TEA005128.1	Scaffold1857:381589:396451:-	265	798	4	PF03936	TPSGD_VITVI, (-)-germacrene D synthase	TPS-a
**9**	CsTPS09	TEA017888.1	Scaffold5016:408273:412431:-	373	1122	5	PF03936	TPSGD_VITVI, (-)-germacrene D synthase	TPS-a
**10**	CsTPS10	TEA032538.1	Scaffold2014:1849901:1856947:-	432	1299	6	PF01397 / PF03936	TPSGD_VITVI, (-)-germacrene D synthase	TPS-a
**11**	CsTPS11	TEA005117.1	Scaffold1857:396807:397525:-	195	588	1	PF01397	TPSGD_VITVI, (-)-germacrene D synthase	TPS-a
**12**	CsTPS12	TEA022784.1	Scaffold1059:445091:449726:-	385	1158	6	PF01397	TPSGD_VITVI, (-)-germacrene D synthase	TPS-a
**13**	CsTPS13	TEA021924.1	Scaffold1039:738385:745242:+	301	906	6	PF03936	TPSGD_VITVI, (-)-germacrene D synthase	TPS-a
**14**	CsTPS14	TEA017878.1	Scaffold5016:412689:414195:-	280	843	3	PF01397	TPSGD_VITVI, (-)-germacrene D synthase	TPS-a
**15**	CsTPS15	TEA002287.1	Scaffold569:2037188:2039624:+	315	948	4	PF01397 / PF03936	TPSGD_VITVI, (-)-germacrene D synthase	TPS-a
**16**	CsTPS16	TEA017309.1	Scaffold4032:135383:138230:-	207	624	3	PF03936	TPSGD_VITVI, (-)-germacrene D synthase	TPS-a
**17**	CsTPS17	TEA033098.1	Scaffold5126:880963:886581:-	185	558	3	PF01397	TPSGD_VITVI, (-)-germacrene D synthase	TPS-a
**18**	CsTPS18	TEA029355.1	Scaffold559:506685:510183:+	207	624	2	PF01397	TPSGD_VITVI, (-)-germacrene D synthase	TPS-a
**19**	CsTPS19	TEA031961.1	Scaffold982:978097:979246:+	186	561	2	PF01397	TPSGD_VITVI, (-)-germacrene D synthase	TPS-a

**20**	CsTPS20	TEA029353.1	Scaffold559:545565:545945:+	126	381	1	PF03936	TPSGD_VITVI, (-)-germacrene D synthase	TPS-a
**21**	CsTPS21	TEA029348.1	Scaffold559:382474:391211:+	730	2193	10	PF01397 / PF03936	TPSGD_VITVI, (-)-germacrene D synthase	TPS-a
**22**	CsTPS22	TEA012463.1	Scaffold1840:218881:222925:-	547	1644	7	PF01397 / PF03936	TPSGD_VITVI, (-)-germacrene D synthase	TPS-a
**23**	CsTPS23	TEA023168.1	Scaffold485:499764:506329:+	540	1623	7	PF01397 / PF03936	TPSGD_VITVI, (-)-germacrene D synthase	TPS-a
**24**	CsTPS24	TEA017886.1	Scaffold5016:315868:326381:-	527	1584	7	PF01397 / PF03936	TPSGD_VITVI, (-)-germacrene D synthase	TPS-a
**25**	CsTPS25	TEA010551.1	Scaffold1188:1285004:1291162:-	839	2520	7	PF01397 / PF03936	TPSGD_VITVI, (-)-germacrene D synthase	TPS-a
**26**	CsTPS26	TEA003774.1	Scaffold5587:3985:6273:-	367	1104	5	PF03936	TPSGD_VITVI, (-)-germacrene D synthase	TPS-a
**27**	CsTPS27	TEA005096.1	Scaffold1857:406724:409024:-	131	396	2	PF01397	TPSGD_VITVI, (-)-germacrene D synthase	TPS-a
**28**	CsTPS28	TEA005082.1	Scaffold10509:51897:63536:-	399	1356	6	PF01397 / PF03936	TPSGD_VITVI, (-)-germacrene D synthase	TPS-a
**29**	CsTPS29	TEA024081.1	Scaffold1689:579330:586136:-	541	1626	7	PF01397 / PF03936	TPS32_SOLLC, Viridiflorene synthase	TPS-a
**30**	CsTPS30	TEA007878.1	Scaffold23:868027:874480:+	487	1464	6	PF01397 / PF03936	TPS32_SOLLC, Viridiflorene synthase	TPS-a
**31**	CsTPS31	TEA027025.1	Scaffold228:1416204:1431743:-	338	1017	5	PF01397	TPS32_SOLLC, Viridiflorene synthase	TPS-a
**32**	CsTPS32	TEA021499.1	Scaffold626:560395:570357:+	435	1308	7	PF01397 / PF03936	TPS32_SOLLC, Viridiflorene synthase	TPS-a
**33**	CsTPS33	TEA010904.1	Scaffold1955:1784442:1785892:+	285	855	5	PF01397 / PF03936	TPSVS_VITVI, Valencene synthase	TPS-a
**34**	CsTPS34	TEA017307.1	Scaffold4032:138287:144440:-	223	672	3	PF01397 / PF03936	TPSVS_VITVI, Valencene synthase	TPS-a
**35**	CsTPS35	TEA027070.1	Scaffold228:1596311:1599132:-	186	561	5	PF01397	5EAS4_NICAT, Probable 5-epi-aristolochene synthase 4	TPS-a
**36**	CsTPS36	TEA027061.1	Scaffold228:1547137:1548048:-	174	525	3	PF03936	TPS1_RICCO, Alpha-copaene synthase	TPS-a
**37**	CsTPS37	TEA009449.1	Scaffold1786:1345647:1360438:-	595	1788	7	PF01397 / PF03936	ATESY_VITVI, (-)-alpha-terpineol synthase	TPS-b
**38**	CsTPS38	TEA023157.1	Scaffold2616:240593:250372:-	500	1503	6	PF01397 / PF03936	ATESY_VITVI, (-)-alpha-terpineol synthase	TPS-b
**39**	CsTPS39	TEA009450.1	Scaffold1786:1406070:1450966:-	236	711	3	PF03936	TPS10_RICCO, Terpene synthase 10	TPS-b
**40**	CsTPS40	TEA017654.1	Scaffold285:1744284:1744859:+	153	462	3	PF01397	ATESY_VITVI, (-)-alpha-terpineol synthase	TPS-b
**41**	CsTPS41	TEA032709.1	Scaffold1618:391002:404626:+	599	1800	8	PF01397 / PF03936	ATESY_VITVI, (-)-alpha-terpineol synthase	TPS-b

**42**	CsTPS42	TEA002963.1	Scaffold2253:770954:781331:+	568	1707	7	PF01397 / PF03936	ATESY_VITVI, (-)-alpha-terpineol synthase	TPS-b
**43**	CsTPS43	TEA014987.1	Scaffold5359:390672:398897:+	607	1824	7	PF01397 / PF03936	TPS9_RICCO, Probable terpene synthase 9	TPS-b
**44**	CsTPS44	TEA032731.1	Scaffold1618:216308:232484:+	583	1752	7	PF01397 / PF03936	ATESY_VITVI, (-)-alpha-terpineol synthase	TPS-b
**45**	CsTPS45	TEA030379.1	Scaffold300:536240:539893:-	473	1472	6	PF01397 / PF03936	ATESY_VITVI, (-)-alpha-terpineol synthase	TPS-b
**46**	CsTPS46	TEA020730.1	Scaffold281:2892997:2893962:+	243	732	3	PF01397 / PF03936	ATESY_VITVI, (-)-alpha-terpineol synthase	TPS-b
**47**	CsTPS47	TEA022294.1	Scaffold881:746241:773646:-	547	1644	9	PF01397 / PF03936	ATESY_VITVI, (-)-alpha-terpineol synthase	TPS-b
**48**	CsTPS48	TEA014219.1	Scaffold2428:561525:589292:+	558	1677	8	PF01397 / PF03936	ATESY_VITVI, (-)-alpha-terpineol synthase	TPS-b
**49**	CsTPS49	TEA023784.1	Scaffold4432:595162:597357:-	303	912	4	PF01397 / PF03936	ATESY_VITVI, (-)-alpha-terpineol synthase	TPS-b
**50**	CsTPS50	TEA007029.1	Scaffold4476:460151:468825:-	390	1173	5	PF01397 / PF03936	ATESY_VITVI, (-)-alpha-terpineol synthase	TPS-b
**51**	CsTPS51	TEA033306.1	Scaffold596:223621:233875:+	528	1587	7	PF01397 / PF03936	TPS7_RICCO, Alpha-farnesene synthase	TPS-b
**52**	CsTPS52	TEA009170.1	Scaffold1816:859236:866711:+	454	1365	7	PF01397 / PF03936	TPS7_RICCO, Alpha-farnesene synthase	TPS-b
**53**	CsTPS53	TEA026271.1	Scaffold3165:1751525:1755140:-	334	1005	5	PF01397 / PF03936	TPS7_RICCO, Alpha-farnesene synthase	TPS-b
**54**	CsTPS54	TEA012490.1	Scaffold6781:57499:58656:-	188	567	2	PF03936	AFS1_MALDO, (E,E)-alpha-farnesene synthase	TPS-b
**55**	CsTPS55	TEA009866.1	Scaffold4688:2601:3839:-	182	549	2	PF03936	AFS1_MALDO, (E,E)-alpha-farnesene synthase	TPS-b
**56**	CsTPS56	TEA004653.1	Scaffold3940:666045:676363:+	136	411	2	PF03936	TPS7_RICCO, Alpha-farnesene synthase	TPS-b
**57**	CsTPS57	TEA004606.1	Scaffold10585:153621:173467:-	521	1566	7	PF01397 / PF03936	SAUBS_SANAS, Beta-bisabolene synthase	TPS-b
**58**	CsTPS58	TEA031754.1	Scaffold2326:935654:955868:+	560	1683	8	PF01397 / PF03936	SAUBS_SANAS, Beta-bisabolene synthase	TPS-b
**59**	CsTPS59	TEA026152.1	Scaffold11662:400841:404486:+	188	567	3	PF01397	SAUBS_SANAS, Beta-bisabolene synthase	TPS-b
**60**	CsTPS60	TEA014989.1	Scaffold5359:1062848:1075257:-	451	1356	6	PF01397 / PF03936	TPS2_LOTJA, Tricyclene synthase EBOS, chloroplastic	TPS-b
**61**	CsTPS61	TEA014996.1	Scaffold5359:1134064:1143428:-	371	1116	7	PF01397 / PF03936	TPS4_MEDTR, Tricyclene synthase TPS4, chloroplastic	TPS-b
**62**	CsTPS62	TEA016812.1	Scaffold4385:22190:22783:+	150	477	2	PF03936	TPS4_MEDTR, Tricyclene synthase TPS4, chloroplastic	TPS-b
**63**	CsTPS63	TEA031457.1	Scaffold1399:777225:788144:-	500	1500	7	PF01397 / PF03936	TPS12_RICCO, Probable terpene synthase 12	TPS-b

**64**	CsTPS64	TEA031444.1	Scaffold1399:767017:769790:-	430	1293	6	PF03936	ISPS_POPTM, Isoprene synthase, chloroplastic	TPS-b
**65**	CsTPS65	TEA031453.1	Scaffold1399:675106:678765:-	398	1197	6	PF01397 / PF03936	ISPS_POPTM, Isoprene synthase, chloroplastic	TPS-b
**66**	CsTPS66	TEA020739.1	Scaffold281:320490:329627:-	296	891	6	PF01397 / PF03936	ATESY_MAGGA, Alpha-terpineol synthase, chloroplastic	TPS-b
**67**	CsTPS67	TEA000496.1	Scaffold682:1760053:1779446:+	723	2172	11	PF01397 / PF03936	KSA_PEA, Ent-copalyl diphosphate synthase, chloroplastic	TPS-c
**68**	CsTPS68	TEA024173.1	Scaffold2697:535837:540208:+	274	825	5	PF03936	KSB_ARATH, Ent-kaur-16-ene synthase, chloroplastic	TPS-e
**69**	CsTPS69	TEA017740.1	Scaffold1561:239440:241090:-	297	894	6	PF01397 / PF03936	KSB_ARATH, Ent-kaur-16-ene synthase, chloroplastic	TPS-e
**70**	CsTPS70	TEA024176.1	Scaffold2697:527999:534944:+	454	1365	7	PF01397 / PF03936	KSB_CUCMA, Ent-kaur-16-ene synthase, chloroplastic	TPS-e
**71**	CsTPS71	TEA008638.1	Scaffold217:754423:765133:-	202	609	3	PF03936	KSB_CUCMA, Ent-kaur-16-ene synthase, chloroplastic	TPS-e
**72**	CsTPS72	TEA007033.1	Scaffold4476:490748:497232:-	426	1281	9	PF03936	ATESY_VITVI, (-)-alpha-terpineol synthase	TPS-e
**73**	CsTPS73	TEA019347.1	Scaffold1889:780853:787842:-	801	2430	13	PF01397 / PF03936	GES_ARATH, (E,E)-geranyllinalool synthase	TPS-f
**74**	CsTPS74	TEA005451.1	Scaffold1133:280712:284333:-	565	1698	9	PF01397 / PF03936	GES_ARATH, (E,E)-geranyllinalool synthase	TPS-f
**75**	CsTPS75	TEA005162.1	Scaffold5444:588299:592626:-	500	1503	7	PF03936	GES_ARATH, (E,E)-geranyllinalool synthase	TPS-f
**76**	CsTPS76	TEA007191.1	Scaffold4183:109141:116548:-	557	1674	7	PF01397 / PF03936	NES1_FRAAN, (3S,6E)-nerolidol synthase 1	TPS-g
**77**	CsTPS77	TEA004822.1	Scaffold1497:1131549:1136588:+	557	1674	7	PF01397 / PF03936	NES1_FRAAN, (3S,6E)-nerolidol synthase 1	TPS-g
**78**	CsTPS78	TEA019472.1	Scaffold161:572157:579727:+	534	1605	7	PF01397 / PF03936	NES1_FRAAN, (3S,6E)-nerolidol synthase 1	TPS-g
**79**	CsTPS79	TEA004657.1	Scaffold3940:831501:836109:+	598	1797	7	PF01397 / PF03936	TPS13_RICCO, Probable terpene synthase 13	TPS-g
**80**	CsTPS80	TEA018721.1	Scaffold3180:769431:773238:-	450	1356	7	PF01397 / PF03936	TPS13_RICCO, Probable terpene synthase 13	TPS-g

Supplementary Table S3. Identification of tea TPS gene family in CSA genome

CSA					Blast against CSS				
**NO.**	**Gene ID**	**Predicted protein(aa)**	**SWISS PROT(HMMER)**	**Pfam**	**Gene ID**	**Predicted protein(aa)**	**Protein Similarity**	**CDS similarity**	**Coverage (nucleotide)**
**1**	CSA000384.1	433	LINS_ARATH S-(+)-linalool synthase, chloroplastic	PF03936	TEA004657.1	598	99%	99%	100%
**2**	CSA012738.1	103	TPSGD_VITVI, (-)-germacrene D synthase	PF01397	TEA029355.1	207	100%	100%	91%
**3**	CSA009231.1	213	TPSGD_VITVI, (-)-germacrene D synthase	PF01397	TEA032539.1	567	99%	99%	99%
**4***	CSA006772.1	544	TPSGD_VITVI, (-)-germacrene D synthase	PF03936/PF01397	TEA014184.1	569	99%	99%	93%
**5**	CSA019627.1	166	RLC1_CITSI, (R)-limonene synthase 1, chloroplastic	PF01397	TEA023157.1	500	98%	98%	100%
**6**	CSA025505.1	250	VTSS2_HYOMU, Vetispiradiene synthase 2 (Fragment)	PF03936	TEA024081.1	541	99%	99%	100%
**7**	CSA035357.1	147	TPSGD_VITVI, (-)-germacrene D synthase	PF03936	TEA010904.1	284	99%	99%	100%
**8**	CSA018887.1	185	TPS10_RICCO, Terpene synthase 10	PF03936	TEA020730.1	243	99%	99%	96%
**9**	CSA019813.1	151	TPS12_RICCO, Probable terpene synthase 12	PF03936	TEA014989.1	451	99%	99%	92%
**10**	CSA009230.1	166	TPSGD_VITVI, (-)-germacrene D synthase	PF03936	TEA032539.1	567	98%	98%	100%
**11**	CSA022343.1	413	ATESY_VITVI, (-)-alpha-terpineol synthase	PF03936/PF01397	TEA009449.1	595	98%	98%	100%
**12**	CSA026804.1	519	TPSGD_VITVI, (-)-germacrene D synthase	PF03936/PF01397	TEA012463.1	547	98%	98%	100%
**13**	CSA016735.1	482	TPS13_RICCO, Probable terpene synthase 13	PF03936/PF01397	TEA004657.1	598	98%	98%	100%
**14**	CSA018886.1	96	ATESY_VITVI, (-)-alpha-terpineol synthase	PF01397	TEA032731.1	583	98%	98%	96%
**15**	CSA019812.1	130	ATESY_MAGGA, Alpha-terpineol synthase, chloroplastic	PF01397	TEA014996.1	371	98%	98%	92%
**16**	CSA005676.1	101	TPSGD_VITVI, (-)-germacrene D synthase	PF03936	TEA031966.1	441	96%	96%	96%
**17**	CSA026262.1	84	LABER_LAVAN, Exo-alpha-bergamotene synthase	PF01397	TEA007033.1	426	96%	96%	98%
**18**	CSA025571.1	213	TPSGD_VITVI, (-)-germacrene D synthase	PF01397	TEA029348.1	730	95%	95%	99%
**19**	CSA025572.1	169	TPSGD_VITVI, (-)-germacrene D synthase	PF03936	TEA029356.1	537	96%	96%	97%
**20**	CSA014761.1	63	ATESY_VITVI, (-)-alpha-terpineol synthase	PF01397	TEA030379.1	473	93%	93%	100%
**21**	CSA020011.1	281	TPS9_RICCO, Probable terpene synthase 9	PF03936/PF01397	TEA014987.1	607	99%	99%	97%
**22**	CSA018130.1	75	ISPS_POPCN, Isoprene synthase, chloroplastic	PF01397	TEA014989.1	451	98%	98%	90%
**23**	CSA029044.1	238	NES1_FRAAN, (3S,6E)-nerolidol synthase 1	PF01397	TEA019472.1	534	95%	95%	91%
**24**	CSA034089.1	411	TPSGD_VITVI, (-)-germacrene D synthase	PF03936/PF01397	TEA029356.1	537	95%	95%	94%
**25**	CSA025246.1	127	ATESY_VITVI, (-)-alpha-terpineol synthase	PF01397	TEA030379.1	473	95%	95%	91%
**26**	CSA005369.1	335	NES1_FRAAN, (3S,6E)-nerolidol synthase	PF01397	TEA004822.1	557	98%	98%	90%
**27**	CSA008212.1	600	ATESY_VITVI, (-)-alpha-terpineol synthase	PF03936/PF01397	TEA030379.1	473	95%	95%	99%
**28**	CSA005041.1	114	ATESY_VITVI, (-)-alpha-terpineol synthase	PF01397	TEA017654.1	153	97%	97%	93%
**29**	CSA001660.1	366	ATESY_VITVI, (-)-alpha-terpineol synthase	PF03936	TEA009449.1	595	84%	84%	100%
**30**	CSA001659.1	63	LABER_LAVAN, Exo-alpha-bergamotene synthase	PF01397	TEA030379.1	473	99%	99%	88%
**31**	CSA003199.1	145	ATESY_VITVI, (-)-alpha-terpineol synthase	PF01397	TEA030379.1	473	98%	98%	88%

**32**	CSA033064.1	145	ATESY_VITVI, (-)-alpha-terpineol synthase	PF01397	TEA030379.1	473	98%	98%	88%
**33**	CSA032703.1	157	NES2_FRAAN, (3S,6E)-nerolidol synthase 2	PF03936	TEA007191.1	557	83%	83%	87%
**34**	CSA032320.1	140	TPS10_RICCO, Terpene synthase 10	PF01397	TEA017654.1	153	93%	93%	86%
**35**	CSA003009.1	223	NES1_FRAAN, (3S,6E)-nerolidol synthase	PF03936	TEA004657.1	598	91%	91%	85%
**36**	CSA014760.1	66	LABER_LAVAN, Exo-alpha-bergamotene synthase	PF01397	TEA030379.1	473	99%	99%	84%
**37**	CSA032393.1	173	ATESY_VITVI, (-)-alpha-terpineol synthase	PF01397	TEA007033.1	426	98%	98%	84%
**38**	CSA005131.1	165	TPSGD_VITVI, (-)-germacrene D synthase	PF01397	TEA032539.1	567	92%	92%	83%
**39**	CSA018895.1	296	TPSGD_VITVI, (-)-germacrene D synthase	PF03936	TEA029348.1	730	97%	97%	83%
**40**	CSA020936.1	502	NES1_FRAAN, (3S,6E)-nerolidol synthase 1	PF03936/PF01397	TEA019472.1	534	99%	99%	83%
**41**	CSA005675.1	299	TPSGD_VITVI, (-)-germacrene D synthase	PF03936	TEA017888.1	373	99%	99%	82%
**42**	CSA033119.1	294	ATESY_VITVI, (-)-alpha-terpineol synthase	PF01397	TEA030379.1	473	77%	77%	82%
**43**	CSA003008.1	229	NES1_FRAAN, (3S,6E)-nerolidol synthase	PF01397	TEA004657.1	598	99%	99%	80%
**44**	CSA012357.1	256	NES1_FRAAN, (3S,6E)-nerolidol synthase 1	PF03936	TEA004657.1	598	98%	98%	78%
**45**	CSA030979.1	316	ATESY_VITVI, (-)-alpha-terpineol synthase	PF03936	TEA007029.1	390	91%	91%	77%
**46**	CSA003485.1	99	SPIBS_SANSP, Alpha-bisabolol synthase	PF01397	TEA007033.1	426	97%	97%	76%
**47**	CSA029126.1	80	ATESY_VITVI, (-)-alpha-terpineol synthase	PF01397	TEA002963.1	568	95%	95%	76%
**48**	CSA022871.1	108	ATESY_VITVI, (-)-alpha-terpineol synthase	PF01397	TEA030379.1	473	92%	92%	75%
**49**	CSA014975.1	116	LABER_LAVAN, Exo-alpha-bergamotene synthase	PF01397	TEA007033.1	426	96%	96%	73%
**50**	CSA019490.1	808	NES1_FRAVE, (3S,6E)-nerolidol synthase 1, chloroplastic	PF03936/PF01397	TEA004657.1	598	96%	96%	69%
**51**	CSA019811.1	98	ISPS_POPTM, Isoprene synthase, chloroplastic	PF01397	TEA014989.1	451	100%	100%	69%
**52**	CSA004219.1	262	TPSGD_VITVI, (-)-germacrene D synthase	PF01397	TEA022784.1	385	100%	100%	68%
**53**	CSA036842.1	843	KSA_PEA, Ent-copalyl diphosphate synthase	PF03936/PF01397	TEA000496.1	723	99%	99%	66%
**54**	CSA036823.1	513	LIS_CLABR, S-linalool synthase	PF03936	TEA005451.1	565	82%	82%	64%
**55**	CSA018888.1	169	ATESY_VITVI, (-)-alpha-terpineol synthase	PF03936	TEA023784.1	303	98%	98%	59%
**56**	CSA022342.1	164	ATESY_VITVI, (-)-alpha-terpineol synthase	PF01397	TEA009449.1	595	100%	100%	54%
**57**	CSA030850.1	138	ATESY_VITVI, (-)-alpha-terpineol synthase	PF01397	TEA007033.1	426	94%	94%	52%
**58**	CSA029009.1	209	TPSGD_VITVI, (-)-germacrene D synthase	PF03936	TEA014184.1	569	98%	98%	49%
**59**	CSA011050.1	202	TPS10_RICCO, Terpene synthase 10	PF01397	TEA014219.1	558	99%	99%	48%
**60**	CSA004334.1	287	TPSGD_VITVI, (-)-germacrene D synthase	PF03936/PF01397	TEA021924.1	301	93%	93%	26%
			Average				96%	96%	84%

*, the highlighted eight TPS genes from both "Yunkang10" and "Shu-Cha-Zao" share similar protein sequence length (90-110%), high identity at both cDNA and protein levels (>90%).

Supplementary Table S4. Identification of putative functional tea TPS gene

Identified in genome							Verified in the full-length transcripts			
**Name**	**Gene ID**	**Protein(aa)**	**exons**	**DDXXD**	**NSE/DTE**	**RRX8W**	**Transcripts ID**	**coverage/identity**	**Reference**	**Annotation**
CsTPS01	TEA032539.1	567	7	YES	YES	YES	CssPBTrans029702/PB.8683.1	1702/1704 (99%)	Xia et al.,2019/ Qiao et al.,2019	full
CsTPS02	TEA029356.1	537	7	YES	YES	YES	CssPBTrans041118/PB.24310.30	1462/1480 (99%)	Xia et al.,2019/ Qiao et al.,2019	full
CsTPS03	TEA014184.1	569	7	YES	YES	NO	CssPBTrans068400/PB.13101.1	1592/1593(99%)	Xia et al.,2019/ Qiao et al.,2019	full
CsTPS04	TEA031969.1	1042	8	YES	YES	YES	PB.31351.2	1653/1682(98%)	Qiao et al.,2019	full
CsTPS05	TEA031966.1	441	5	NO	NO	NO	PB.31351.8	1281/1306(98%)	Qiao et al.,2019	full
CsTPS21	TEA029348.1	730	10	YES	YES	YES	CssPBTrans020115	1645/1648 (99%)	Xia et al.,2019	full
CsTPS22	TEA012463.1	547	7	YES	YES	NO	PB.22660.1	1535/1571(98%)	Qiao et al.,2019	full
CsTPS23	TEA023168.1	540	7	YES	YES	YES	CssPBTrans024950	1649/1649(100%)	Xia et al.,2019	full
CsTPS25	TEA010551.1	839	7	YES	YES	NO	CssPBTrans026737/PB.1889.1	1369/1397 (98%)	Xia et al.,2019/ Qiao et al.,2019	full
CsTPS29	TEA024081.1	541	7	YES	NO	NO	No hits found			full
CsTPS42	TEA002963.1	568	7	YES	YES	YES	CssPBTrans058925	1704/1707 (99%)	Xia et al.,2019	full
CsTPS43	TEA014987.1	607	7	YES	YES	YES	No hits found			full
CsTPS45	TEA030379.1	473	6	YES	NO	YES	CssPBTrans048789	1098/1123 (98%)	Xia et al.,2019	full
CsTPS47	TEA022294.1	547	8	NO	YES	NO	PB.21690.2	1004/1007(99%)	Qiao et al.,2019	full
CsTPS51	TEA033306.1	528	7	YES	YES	NO	PB.25018.1	1251/1265(99%)	Qiao et al.,2019	full
CsTPS57	TEA004606.1	521	7	YES	YES	NO	CssPBTrans046146/PB.522.1	1517/1536 (99%)	Xia et al.,2019/ Qiao et al.,2019	full
CsTPS63	TEA031457.1	500	7	YES	YES	NO	PB.3574.1	1494/1503(99%)	Qiao et al.,2019	full
CsTPS70	TEA024176.1	454	7	YES	NO	NO	CssPBTrans055745	1290/1308(98%)	Xia et al.,2019	full
CsTPS73	TEA019347.1	801	13	YES	NO	NO	No hits found			full
CsTPS76	TEA007191.1	557	7	YES	YES	NO	PB.20489.1	1517/1530 (99%)	Qiao et al.,2019	full
CsTPS77	TEA004822.1	557	7	YES	YES	NO	PB.4391.1	1528/1530 (99%)	Qiao et al.,2019	full
CsTPS78	TEA019472.1	534	7	YES	YES	NO	PB.5304.3	1538/1546(99%)	Qiao et al.,2019	full
CsTPS79	TEA004657.1	598	7	YES	YES	NO	PB.19331.4	1516/1547 (98%)	Qiao et al.,2019	full

Identified in genome							Verified in the full-length transcripts			
**Name**	**Gene ID**	**Protein(aa)**	**exons**	**DDXXD**	**NSE/DTE**	**RRX8W**	**Transcripts ID**	**coverage/identity**	**Reference**	**Annotation**
CsTPS06	TEA025177.1	624	9	YES	YES	YES	CssPBTrans041118/PB.31351.8	1077/1145 (94%)	Xia et al.,2019/ Qiao et al.,2019	full?
CsTPS07	TEA010895.1	559	8	YES	YES	NO	CssPBTrans041118/PB.24310.21	1287/1361 (95%)	Xia et al.,2019/ Qiao et al.,2019	full?
CsTPS24	TEA017886.1	527	7	YES	YES	NO	CssPBTrans024950/PB.22660.1	1180/1230 (95%)	Xia et al.,2019/ Qiao et al.,2019	full?
CsTPS37	TEA009449.1	595	7	YES	YES	NO	CssPBTrans048789	1328/1555 (85%)	Xia et al.,2019	full?
CsTPS41	TEA032709.1	599	8	YES	YES	YES	CssPBTrans058925	639/648 (99%)	Xia et al.,2019	full?
CsTPS44	TEA032731.1	583	7	YES	NO	NO	CssPBTrans058925	639/648 (99%)	Xia et al.,2019	full?

CsTPS48	TEA014219.1	558	8	NO	YES	NO	PB.21690.2	585/599(98%)	Qiao et al.,2019	full?
CsTPS58	TEA031754.1	560	8	YES	YES	NO	CssPBTrans026173/PB.522.2	850/851（99%）	Xia et al.,2019/ Qiao et al.,2019	full?
CsTPS67	TEA000496.1	723	11	NO	NO	NO	CssPBTrans065569/PB.30438.1	482/485（99%）	Xia et al.,2019/ Qiao et al.,2019	full?

**Identified in genome**							**Verified in the full-length transcripts**			
**Name**	**Gene ID**	**Protein(aa)**	**exons**	**DDXXD**	**NSE/DTE**	**RRX8W**	**Transcripts ID**	**coverage/identity**	**Reference**	**Annotation**
CsTPS08	TEA005128.1	265	4	NO	NO	NO	CssPBTrans029702/PB.24310.11	769/798（96%）	Xia et al.,2019/ Qiao et al.,2019	partial
CsTPS09	TEA017888.1	373	5	NO	NO	NO	CssPBTrans029702/PB.31351.8	683/741（92%）	Xia et al.,2019/ Qiao et al.,2019	partial
CsTPS10	TEA032538.1	432	6	YES	NO	NO	CssPBTrans057668/PB.24310.16	727/767（95%）	Xia et al.,2019/ Qiao et al.,2019	partial
CsTPS11	TEA005117.1	195	1	NO	NO	NO	CssPBTrans057668/PB.24310.21	353/355（99%）	Xia et al.,2019/ Qiao et al.,2019	partial
CsTPS12	TEA022784.1	385	6	NO	NO	NO	CssPBTrans033837/PB.13101.1	560/584 (96%)	Xia et al.,2019/ Qiao et al.,2019	partial
CsTPS13	TEA021924.1	301	6	NO	YES	NO	CssPBTrans029702	280/313（89%）	Xia et al.,2019	partial
CsTPS14	TEA017878.1	280	3	NO	NO	YES	CssPBTrans078631/PB.8683.4	367/378（97%）	Xia et al.,2019/ Qiao et al.,2019	partial
CsTPS15	TEA002287.1	315	4	YES	NO	YES	CssPBTrans049906/PB.24310.23	390/404（97%）	Xia et al.,2019/ Qiao et al.,2019	partial
CsTPS16	TEA017309.1	207	3	NO	NO	NO	CssPBTrans046143/PB.31351.8	523/601（87%）	Xia et al.,2019/ Qiao et al.,2019	partial
CsTPS17	TEA033098.1	185	3	NO	NO	NO	CssPBTrans070005/PB.18777.3	82/83（99%）	Xia et al.,2019/ Qiao et al.,2019	partial
CsTPS18	TEA029355.1	207	2	NO	NO	NO	CssPBTrans072461	273/286（95%）	Xia et al.,2019	partial
CsTPS19	TEA031961.1	186	2	NO	NO	YES	CssPBTrans036197/PB.31351.2	402/434（93%）	Xia et al.,2019/ Qiao et al.,2019	partial
CsTPS20	TEA029353.1	126	1	NO	NO	NO	CssPBTrans072461/PB.24310.26	359/378（95%）	Xia et al.,2019/ Qiao et al.,2019	partial
CsTPS26	TEA003774.1	367	5	YES	NO	NO	CssPBTrans024950/PB.22660.1	797/817（98%）	Xia et al.,2019/ Qiao et al.,2019	partial
CsTPS27	TEA005096.1	131	2	NO	NO	NO	CssPBTrans026737/PB.22660.1	191/199（96%）	Xia et al.,2019/ Qiao et al.,2019	partial
CsTPS28	TEA005082.1	399	6	NO	NO	NO	CssPBTrans032981/PB.31165.4	643/677（95%）	Xia et al.,2019/ Qiao et al.,2019	partial
CsTPS30	TEA007878.1	487	6	NO	NO	NO	CssPBTrans032981	1081/1136（95%）	Xia et al.,2019	partial
CsTPS31	TEA027025.1	338	5	NO	NO	YES	CssPBTrans024114/PB.24110.1	616/647（95%）	Xia et al.,2019/ Qiao et al.,2019	partial
CsTPS32	TEA021499.1	435	7	NO	NO	YES	CssPBTrans032981	396/398（99%）	Xia et al.,2019	partial
CsTPS33	TEA010904.1	285	5	YES	NO	NO	CssPBTrans057668/PB.31351.9	309/322（96%）	Xia et al.,2019/ Qiao et al.,2019	partial

CsTPS34	TEA017307.1	223	3	YES	NO	NO	CssPBTrans072461/PB.24310.26	313/329（95%）	Xia et al.,2019/ Qiao et al.,2019	partial
CsTPS35	TEA027070.1	186	5	NO	NO	NO	CssPBTrans024114	361/380（95%）	Xia et al.,2019	partial
CsTPS36	TEA027061.1	174	3	NO	NO	NO	CssPBTrans032981/PB.4785.11	225/230（98%）	Xia et al.,2019/ Qiao et al.,2019	partial
CsTPS38	TEA023157.1	500	6	YES	NO	YES	No hits found			partial
CsTPS39	TEA009450.1	236	3	NO	YES	YES	CssPBTrans058925	534/572（93%）	Xia et al.,2019	partial
CsTPS40	TEA017654.1	153	3	NO	NO	YES	CssPBTrans058211	266/319（83%）	Xia et al.,2019	partial
CsTPS46	TEA020730.1	243	3	YES	NO	NO	CssPBTrans048789/PB.21573.8	697/716 (97%)	Xia et al.,2019/ Qiao et al.,2019	partial
CsTPS49	TEA023784.1	303	4	YES	NO	NO	CssPBTrans052197/PB.21573.5	345/359（96%）	Xia et al.,2019/ Qiao et al.,2019	partial
CsTPS50	TEA007029.1	390	5	NO	YES	NO	CssPBTrans035865/PB.22991.1	138/148（93%）	Xia et al.,2019/ Qiao et al.,2019	partial
CsTPS52	TEA009170.1	454	7	YES	NO	YES	PB.25018.1	1326/1346(98%)	Qiao et al.,2019	partial
CsTPS53	TEA026271.1	334	5	YES	NO	NO	PB.25018.1	947/963(98%)	Qiao et al.,2019	partial
CsTPS54	TEA012490.1	188	2	NO	YES	NO	PB.7071.2	548/550(99%)	Qiao et al.,2019	partial
CsTPS55	TEA009866.1	182	2	NO	YES	NO	PB.7071.1	99/100(99%)	Qiao et al.,2019	partial
CsTPS56	TEA004653.1	136	2	NO	YES	NO	PB.7071.1	262/262（100%）	Qiao et al.,2019	partial
CsTPS59	TEA026152.1	188	3	NO	NO	NO	CssPBTrans072185/PB.1592.8	595/609（98%）	Xia et al.,2019/ Qiao et al.,2019	partial
CsTPS60	TEA014989.1	451	6	YES	NO	NO	CssPBTrans026167/PB.23856.1	351/362（97%）	Xia et al.,2019/ Qiao et al.,2019	partial
CsTPS61	TEA014996.1	371	7	YES	NO	NO	CssPBTrans026167	351/362（97%）	Xia et al.,2019	partial
CsTPS62	TEA016812.1	150	2	NO	YES	NO	PB.3574.1	349/360(96%)	Qiao et al.,2019	partial
CsTPS64	TEA031444.1	430	6	YES	NO	NO	PB.3574.1	475/505(90%)	Qiao et al.,2019	partial
CsTPS65	TEA031453.1	398	6	YES	NO	NO	No hits found			partial
CsTPS66	TEA020739.1	296	6	YES	NO	NO	CssPBTrans026167/PB.13892.1	322/333（97%）	Xia et al.,2019/ Qiao et al.,2019	partial
CsTPS68	TEA024173.1	274	5	NO	NO	NO	CssPBTrans038300/PB.13128.1	275/294（94%）	Xia et al.,2019/ Qiao et al.,2019	partial
CsTPS69	TEA017740.1	297	6	YES	NO	NO	CssPBTrans056949/PB.13127.2	699/704（99%）	Xia et al.,2019/ Qiao et al.,2019	partial
CsTPS71	TEA008638.1	202	3	NO	NO	NO	CssPBTrans022223/PB.13127.7	419/425（99%）	Xia et al.,2019/ Qiao et al.,2019	partial
CsTPS72	TEA007033.1	426	9	NO	NO	NO	No hits found			partial
CsTPS74	TEA005451.1	565	9	NO	YES	NO	CssPBTrans050478/PB.24009.1	1646/1689（97%）	Xia et al.,2019/ Qiao et al.,2019	partial
CsTPS75	TEA005162.1	500	7	YES	YES	NO	CssPBTrans050478/PB.24009.1	726/736（99%）	Xia et al.,2019/ Qiao et al.,2019	partial
CsTPS80	TEA018721.1	450	7	NO	YES	NO	CssPBTrans038907/PB.19331.5	628/647（97%）	Xia et al.,2019/ Qiao et al.,2019	partial

Supplementary Table S5 Identification of transposable elements in tea TPS genes

Name	gene ID	Gene location	Transposon ID	Location	Class	Number
CsTPS10	TEA032538.1	Scaffold2014:1849901:1856947:-	TE311378	Scaffold2014:1853913:1854085:-	LINE/L1	1
CsTPS13	TEA021924.1	Scaffold1039:738385:745242:+	TE164009	Scaffold1039:744626:744733:+	LTR/Copia	1
CsTPS24	TEA017886.1	Scaffold5016:315868:326381:-	TE546805	Scaffold5016:318934:319633:-	LTR/Copia	1
CsTPS28	TEA005082.1	Scaffold10509:51897:63536:-	TE625680	Scaffold10509:62665:63201:-	LTR/Copia	1
CsTPS35	TEA027070.1	Scaffold228:1596311:1599132:-	TE529858	Scaffold228:1597128:1597212:-	DNA/hAT	1
CsTPS37	TEA009449.1	Scaffold1786:1345647:1360438:-	TE273709	Scaffold1786:1354512:1355716:-	LINE/L1	4
			TE273710	Scaffold1786:1355117:1355781:-	LINE/L1	
			TE273711	Scaffold1786:1355956:1356223:-	LINE/L1	
			TE273712	Scaffold1786:1358305:1358729:-	LINE/L1	
CsTPS39	TEA009450.1	Scaffold1786:1406070:1450966:-	TE273720	Scaffold1786:1407859:1410112:-	LTR/Copia	11
			TE273721	Scaffold1786:1400171:1412454:-	LTR/Copia	
			TE273722	Scaffold1786:1405810:1426929:-	LTR/Gypsy	
			TE273723	Scaffold1786:1407158:1428929:-	LTR/Gypsy	
			TE273724	Scaffold1786:1401045:1441905:-	LTR/Gypsy	
			TE273725	Scaffold1786:1401897:1442588:-	LTR/Gypsy	
			TE273726	Scaffold1786:1402899:1443119:-	LTR/Gypsy	
			TE273727	Scaffold1786:1403567:1444499:-	LTR/Gypsy	
			TE273728	Scaffold1786:1404517:1444957:-	LTR/Gypsy	
			TE273729	Scaffold1786:1405238:1445603:-	LTR/Gypsy	
			TE273730	Scaffold1786:1407440:1447470:-	LTR/Gypsy	
CsTPS41	TEA032709.1	Scaffold1618:391002:404626:+	TE250880	Scaffold1618:395563:398333:+	LTR/Copia	1
CsTPS44	TEA032731.1	Scaffold1618:216308:232484:+	TE250838	Scaffold1618:220936:221017:+	LTR/Copia	5
			TE250839	Scaffold1618:221146:222249:+	LTR/Copia	
			TE250840	Scaffold1618:222352:222567:+	LTR/Copia	
			TE250841	Scaffold1618:222394:222565:+	LTR/Copia	
			TE250842	Scaffold1618:222593:224090:+	LTR/Copia	
CsTPS48	TEA014219.1	Scaffold2428:561525:589292:+	TE359549	Scaffold2428:577278:577358:+	LTR/Copia	4
			TE359550	Scaffold2428:577884:577986:+	LTR/Copia	
			TE359551	Scaffold2428:578397:579225:+	LTR/Copia	
			TE359552	Scaffold2428:579654:581275:+	LTR/Copia	
CsTPS50	TEA007029.1	Scaffold4476:460151:468825:-	TE522179	Scaffold4476:466233:466387:-	LTR/Gypsy	1
CsTPS53	TEA026271.1	Scaffold3165:1751525:1755140:-	TE429535	Scaffold3165:1754459:1754549:-	DNA/PIF-Harbinger	1
CsTPS57	TEA004606.1	Scaffold10585:153621:173467:-	TE626080	Scaffold10585:157007:157243:-	LINE/L1	1
CsTPS58	TEA031754.1	Scaffold2326:935654:955868:+	TE348377	Scaffold2326:951961:952698:+	LINE/L1	2
			TE348378	Scaffold2326:953063:953147:+	LINE/L1	
CsTPS60	TEA014989.1	Scaffold5359:1062848:1075257:-	TE554481	Scaffold5359:1063890:1065716:-	LTR/Copia	4
			TE554482	Scaffold5359:1066183:1066496:-	LTR/Copia	
			TE554483	Scaffold5359:1066771:1066902:-	LTR/Copia	
			TE554484	Scaffold5359:1067345:1067448:-	LTR/Copia	
CsTPS63	TEA031457.1	Scaffold1399:777225:788144:-	TE214393	Scaffold1399:785957:786101:-	LTR/Copia	1

CsTPS67	TEA000496.1	Scaffold682:1760053:1779446:+	TE089287	Scaffold682:1760606:1761229:+	LTR/Gypsy	3
			TE089288	Scaffold682:1761433:1762666:+	LTR/Gypsy	
			TE089289	Scaffold682:1763042:1763096:+	LTR/Gypsy	
CsTPS70	TEA024176.1	Scaffold2697:527999:534944:+	TE389462	Scaffold2697:530047:533222:+	LINE/RTE-BovB	1
CsTPS72	TEA007033.1	Scaffold4476:490748:497232:-	TE522189	Scaffold4476:496274:496317:-	LINE/L1	1

Supplementary Table S6 TPM value of tea TPS genes in transciptome of eight tea organs

Name	Gene ID	Apical bud	Young leaf	Mature leaf	Old leaf	Stem	Flower	Fruit	Root
CsTPS01	TEA032539	31.3278	23.1825	0.918775	0.12187	5.48689	42.9248	0.0639724	0.127303
CsTPS02	TEA029356	77.7795	79.3262	1.30856	0.043525	2.30623	0.903892	0.0274167	0.019585
CsTPS03	TEA014184	0.149306	1.89402	4.79805	0	0.115311	0.0602595	0.438668	0.019585
CsTPS04	TEA031969	10.2933	7.82464	0.49187	0.0870501	0.586166	22.3462	0.630585	0.11751
CsTPS05	TEA031966	0	0.0257107	0.3341	0.05223	1.05702	1.91826	0.776807	0
CsTPS06	TEA025177	0.500613	0.591347	0.0185611	0	0.124921	0	0.0182778	0
CsTPS07	TEA010895	1.83558	1.33696	0.139208	0	0.0480464	0.080346	0	0
CsTPS08	TEA005128	0.0263481	0.0514215	0	0	0	10.3445	0.0913891	0.0587551
CsTPS09	TEA017888.1								
CsTPS10	TEA032538	0	0	0	0	0	0	0	0.0293775
CsTPS11	TEA005117	28.6843	30.9557	2.58928	0	0.701477	0.150649	0.0822502	0.0783401
CsTPS12	TEA022784	0.0263481	0.068562	0.0742445	0	0	0	0	0.0293775
CsTPS13	TEA021924.1	0	0	0	0	0	0	0	0
CsTPS14	TEA017878	21.7547	17.329	0.872372	0	0.509291	0.100432	0.0548335	0
CsTPS15	TEA002287	0.0878269	0.454223	0	0.10446	0.0960927	0.080346	0.1645	0
CsTPS16	TEA017309.1	0	0	0	0	0	0	0	0
CsTPS17	TEA033098	3.22325	2.35682	4.50107	2.41129	4.09355	5.34301	4.79793	2.03684
CsTPS18	TEA029355	44.9586	16.9605	1.03942	0.0783451	0.836007	0.974195	0	0.0783401
CsTPS19	TEA031961	0.0878269	1.01986	0.204172	0	0.201795	0.451946	0.146223	0
CsTPS20	TEA029353	12.6734	13.5838	0	0	0.643821	2.4606	0	0
CsTPS21	TEA029348	4.11908	5.63922	0.194892	0.0783451	0.52851	322.228	0.173639	1.51784
CsTPS22	TEA012463	0.237133	0.171405	0.603236	0.957551	0.595775	0.0703027	0.219334	0
CsTPS23	TEA023168	0.307394	0.0428512	3.18323	8.07825	5.06409	1.31567	0.703696	0
CsTPS24	TEA017886	0.149306	0.111413	0.0742445	0.391725	0.192185	0.040173	0.155361	0.019585
CsTPS25	TEA010551	0.245915	0.145694	0	0.03482	0	0	0	0
CsTPS26	TEA003774	0	0	0	0.774746	0	0	0	0
CsTPS27	TEA005096.1	0	0	0	0	0	0	0	0
CsTPS28	TEA005082	0.0614788	0	0	0	0	2.55098	0	0.0293775
CsTPS29	TEA024081	0	0	0	0	0.0480464	23.7222	0	0.303568
CsTPS30	TEA007878	0	0.0257107	0	0	0	11.3589	0.0274167	0.0293775
CsTPS31	TEA027025	0	0	0	0	0	22.2759	0	0.15668
CsTPS32	TEA021499	0	0.0257107	0	0	0.0576556	17.3447	0.0274167	0.0979251
CsTPS33	TEA010904.1	0	0	0	0	0	0	0	0
CsTPS34	TEA017307	0.254698	0.0171405	0	0	0.0768742	0.0602595	0	0
CsTPS35	TEA027070	0	0	0	0	0	1.84796	0	0
CsTPS36	TEA027061	0	0	0	0	0	67.2596	0.109667	0.881326
CsTPS37	TEA009449	0.0526961	0.582777	1.47561	0.757336	0.230623	0.0200865	0.0456945	1.79203
CsTPS38	TEA023157	0	0	0	0	0	0.0703027	0	1.6941
CsTPS39	TEA009450	0	0	0.0742445	0	4.29534	0	8.01482	0
CsTPS40	TEA017654	0.237133	0	0	0	0	0	0	0

CsTPS41	TEA032709	0	0	0	0	0.66304	0	0.237612	1.08697
CsTPS42	TEA002963	0	0	0.0278417	0	0.201795	0	1.06925	3.11402
CsTPS43	TEA014987	2.44159	0.557066	5.98596	0	21.4479	3.62561	0.0182778	0.176265
CsTPS44	TEA032731	0	0	0	0	0.0480464	0	0.0822502	0.146888
CsTPS45	TEA030379	1.12418	12.0155	34.6072	21.5101	3.99746	0.100432	1.08753	4.72978
CsTPS46	TEA020730	2.45915	27.7333	84.351	57.2528	8.85975	0.451946	0.42039	0.949874
CsTPS47	TEA022294	0.974878	0.137124	0	0.01741	0.211404	0.190822	0	0.421078
CsTPS48	TEA014219	0.632353	0.0942727	0	0	0.182576	0.160692	0	0.23502
CsTPS49	TEA023784	0.904617	7.79035	13.6981	11.6212	1.78732	0.331427	0.0456945	0.0489626
CsTPS50	TEA007029	0.509396	0.325669	0	0	0.144139	0.522249	0	0.323153
CsTPS51	TEA033306	4.44404	1.85974	4.48251	0	1.9699	0	5.82149	0
CsTPS52	TEA009170	3.23203	1.1227	1.82827	0	0.52851	0.0301297	1.87348	0
CsTPS53	TEA026271	1.67749	0.265678	0	0	0.211404	0	0	0
CsTPS54	TEA012490	16.8188	6.71907	16.3895	0	1.13389	0	10.985	0
CsTPS55	TEA009866	10.1177	3.48809	5.79107	0	4.68932	0	8.91958	0
CsTPS56	TEA004653	0.755311	1.21698	0	0	0	0	0	0
CsTPS57	TEA004606	104.347	50.2216	2.32942	0.45266	30.0001	2.35012	11.6795	0.949874
CsTPS58	TEA031754	19.6205	6.04202	0.389783	0.05223	9.0135	0.944065	9.2303	0.166473
CsTPS59	TEA026152	20.3758	7.13902	0.714603	0.1741	13.6644	1.01437	8.06966	0.254605
CsTPS60	TEA014989	7.32476	5.75921	3.71222	4.97926	1.84498	1.4864	1.10581	0.0783401
CsTPS61	TEA014996	0.500613	0.617058	0.937336	0	0.249841	0	0.137084	0
CsTPS62	TEA016812	0.219567	0.128554	0	0	0	0	0	0
CsTPS63	TEA031457	6.92954	5.72493	0.0278417	0.026115	0	0	0	0
CsTPS64	TEA031444	0.606005	0.35995	0.250575	0.15669	0.288278	0.160692	0.246751	0.11751
CsTPS65	TEA031453.1	0	0	0	0	0	0	0	0
CsTPS66	TEA020739	0.13174	0.111413	0	0	0.0960927	0	0	0
CsTPS67	TEA000496	28.008	33.0726	0.380503	0.12187	12.0692	0.100432	0.0365556	5.20962
CsTPS68	TEA024173	0.711398	0.462793	0.631078	0.29597	0.778351	0.331427	0.594029	0.401493
CsTPS69	TEA017740	5.18179	4.58508	6.5892	3.37754	6.77454	2.93263	9.3491	5.15086
CsTPS70	TEA024176	3.09151	3.05101	4.19481	2.10661	5.46768	3.32432	7.50305	4.20099
CsTPS71	TEA008638	7.6585	7.00189	9.51257	5.17948	11.5792	4.4592	16.2398	7.91235
CsTPS72	TEA007033	0.58844	0.145694	0	0	0.153748	0	0.0913891	0.293775
CsTPS73	TEA019347	0	0	0	0	0	0	0.0456945	0
CsTPS74	TEA005451	1.09784	1.44837	3.60086	0.01741	1.22999	21.8642	0	0.137095
CsTPS75	TEA005162	2.71385	2.75105	9.46617	0.026115	3.68035	28.9848	0.0274167	0.254605
CsTPS76	TEA007191	21.4034	41.4543	4.39898	1.01849	2.26779	16.7923	0	0.0881326
CsTPS77	TEA004822	27.3844	55.1753	7.99984	2.91618	4.78542	2.57107	0.0639724	0.146888
CsTPS78	TEA019472	3.53942	3.24812	0.278417	0.513595	5.41002	119.635	0.0822502	0.519003
CsTPS79	TEA004657	69.6028	41.8485	3.97208	5.15336	19.2762	1378.65	12.301	7.53044
CsTPS80	TEA018721	2.90707	3.53951	1.11367	0.609351	0	14.0605	0	0.35253

Supplementary Fig S1. The structure analysis of *CsTPS67* with three retrotransposons inserted in the first intron. (A), loss of 89 amino acids of *CsTPS67* due to the insertion of retrotransposons compared to other *CPS* genes identified in GenBank (B); Phylogenetic analysis of *CsTPS67* with *CPS* genes of other species.

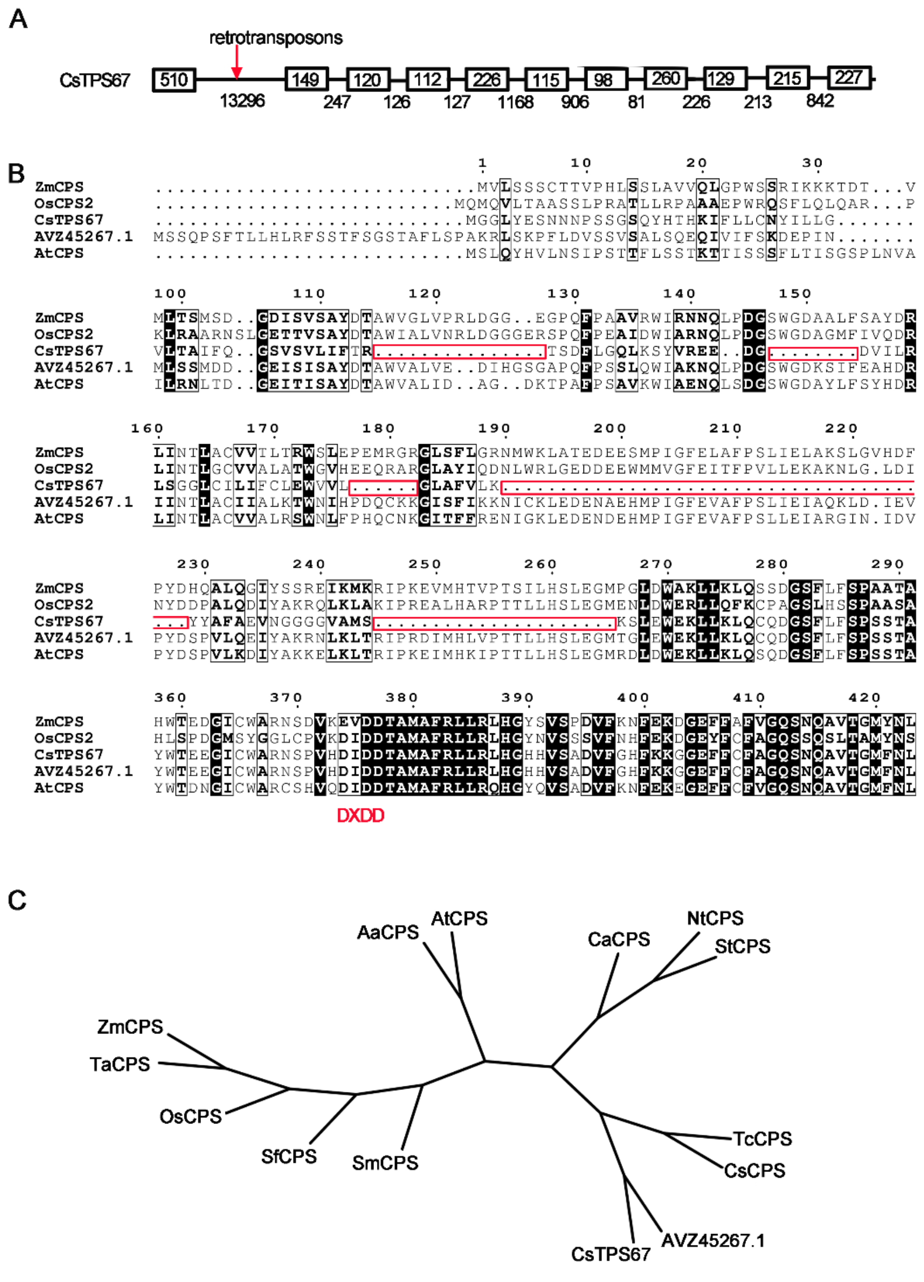

Fig S1

Supplementary Fig S2. Tempo-spatial expression patterns of 80 *CsTPS* genes in tea apical buds (B), young leaves (YL), mature leaves (ML), old leaves (OL), immature stems (S), flowers (FL), young fruits (FR) and roots (R).

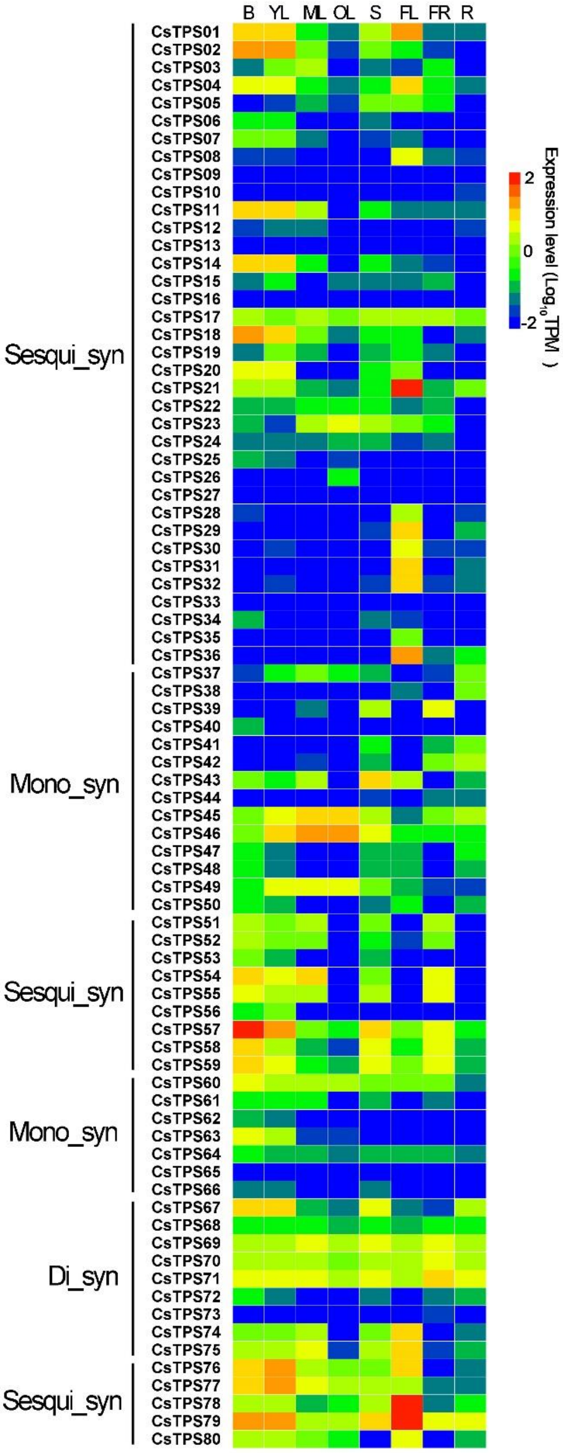

Fig S2

Supplementary Fig S3. Transcriptional responses of all 80 *CsTPS* genes under different abiotic stress conditions.

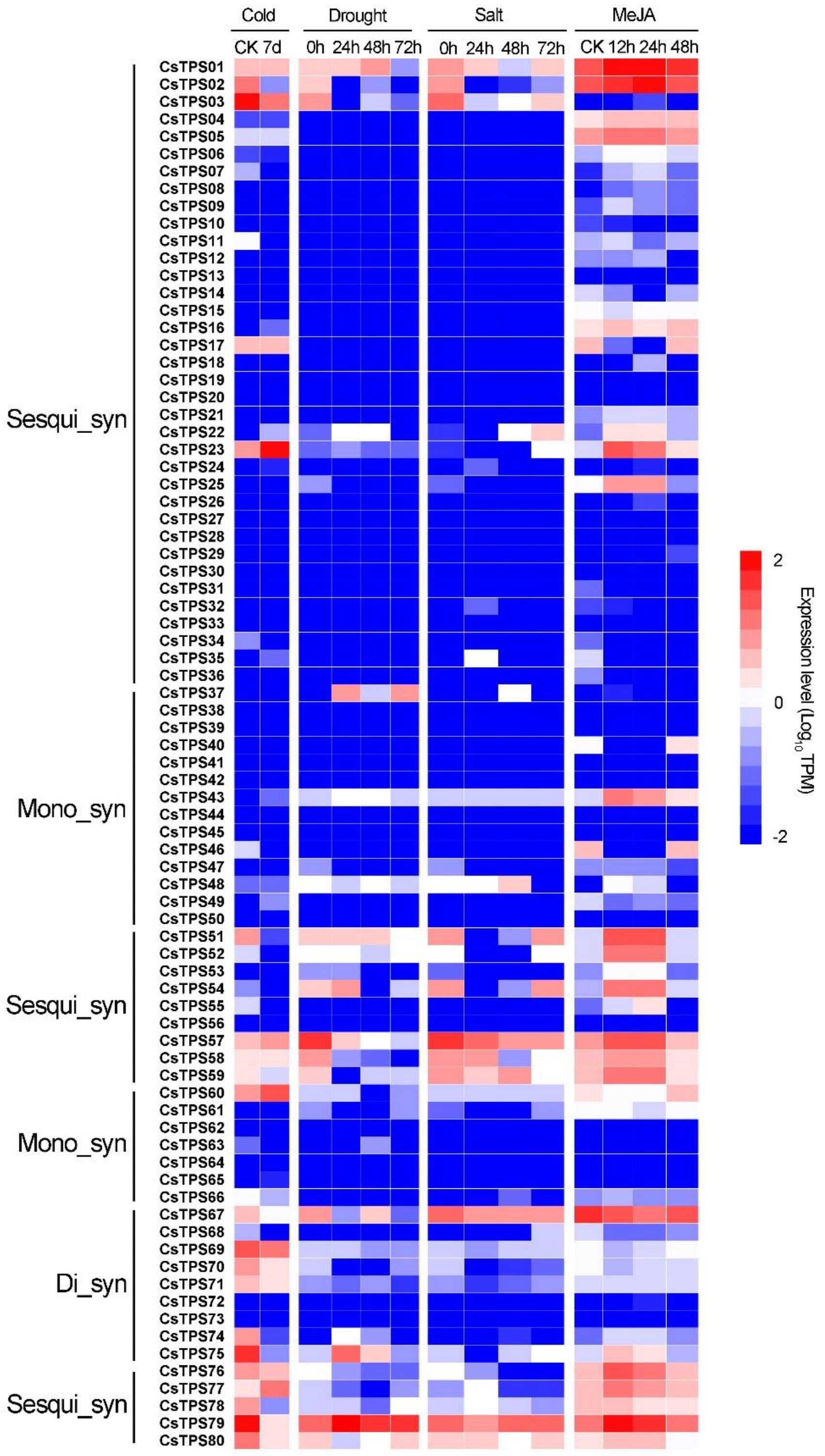

Fig S3
